# Dihydrofolate Reductase Inhibitors: The Pharmacophore as a Guide for Co-Crystal Screening

**DOI:** 10.3390/molecules26216721

**Published:** 2021-11-06

**Authors:** João A. Baptista, Mário T. S. Rosado, Ricardo A. E. Castro, António O. L. Évora, Teresa M. R. Maria, Manuela Ramos Silva, João Canotilho, M. Ermelinda S. Eusébio

**Affiliations:** 1 CQC, Departamento de Química, Universidade de Coimbra,3004-535 Coimbra, Portugal; jabaptista@qui.uc.pt (J.A.B.); mario.rosado@qui.uc.pt (M.T.S.R.); rcastro@ff.uc.pt (R.A.E.C.); antonio.evora@uc.pt (A.O.L.É.); troseiro@ci.uc.pt (T.M.R.M.); jcano@ci.uc.pt (J.C.); 2 Faculdade de Farmácia, Universidade de Coimbra,3000-548 Coimbra, Portugal; 3 Ctr Quim Estrutural, Faculdade Ciências, Universidade de Lisboa,1749-016 Lisboa, Portugal; 4 CfisUC, Departmento de Física, Universidade de Coimbra, 3004-535 Coimbra, Portugal; manuela@fis.uc.pt

**Keywords:** co-crystal screening, dihydrofolate reductase inhibitors, pharmacophore, trimethoprim, pyrimethamine, 2,4-diaminopyrimidine, pyridinecarboxamides, theophylline, caffeine

## Abstract

In this work, co-crystal screening was carried out for two important dihydrofolate reductase (DHFR) inhibitors, trimethoprim (TMP) and pyrimethamine (PMA), and for 2,4-diaminopyrimidine (DAP), which is the pharmacophore of these active pharmaceutical ingredients (API). The isomeric pyridinecarboxamides and two xanthines, theophylline (THEO) and caffeine (CAF), were used as co-formers in the same experimental conditions, in order to evaluate the potential for the pharmacophore to be used as a guide in the screening process. In silico co-crystal screening was carried out using BIOVIA COSMOquick and experimental screening was performed by mechanochemistry and supported by (solid + liquid) binary phase diagrams, infrared spectroscopy (FTIR) and X-ray powder diffraction (XRPD). The in silico prediction of low propensities for DAP, TMP and PMA to co-crystallize with pyridinecarboxamides was confirmed: a successful outcome was only observed for DAP + nicotinamide. Successful synthesis of multicomponent solid forms was achieved for all three target molecules with theophylline, with DAP co-crystals revealing a greater variety of stoichiometries. The crystalline structures of a (1:2) TMP:THEO co-crystal and of a (1:2:1) DAP:THEO:ethyl acetate solvate were solved. This work demonstrated the possible use of the pharmacophore of DHFR inhibitors as a guide for co-crystal screening, recognizing some similar trends in the outcome of association in the solid state and in the molecular aggregation in the co-crystals, characterized by the same supramolecular synthons.

## 1. Introduction

**Figure 1:**
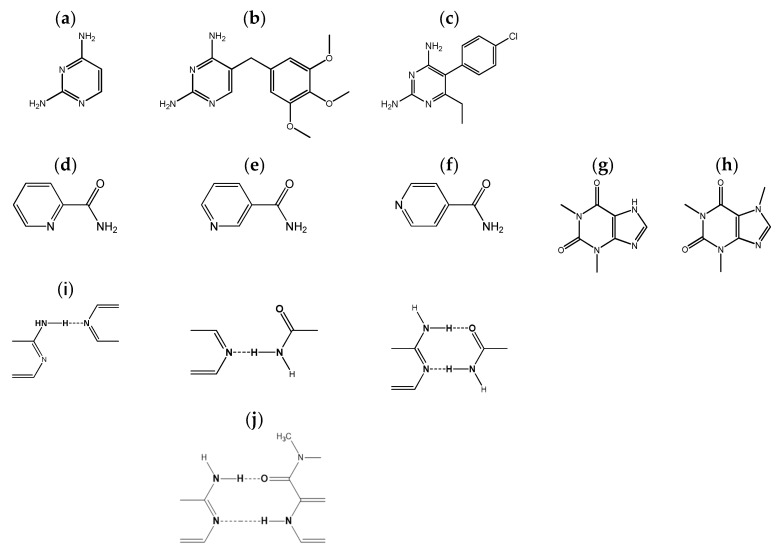
Molecular structures: (**a**) 2,4-diaminopyrimidine, (**b**) trimethoprim, (**c**) pyrimethamine, (**d**) picolinamide, (**e**) nicotinamide, (**f**) isonicotinamide, (**g**) theophylline, (**h**) caffeine. (**i**) Illustration of possible association points between the DAP scaffold and pyridinecarboxamides, (**j**) Illustration of a possible association between the DAP scaffold and theophylline.

Dihydrofolate reductase (DHFR) inhibitors are commonly used as a first-line therapy for diseases such as malaria, tuberculosis and toxoplasmosis [1,2,3,4,5,6,7]. They have also been used, for instance, in cancer therapy [3,8,9,10] and have been investigated as inhibitors of *Bacillus anthracis*, the agent responsible for anthrax [11]. Most DHFR inhibitors share a common 2,4-diaminopyrimidine (DAP) scaffold (Figure 1a.), such as in trimethoprim (TMP) (Figure 1b) and in pyrimethamine (PMA) (Figure 1c), which has been found to play an important role in interactions with the target enzyme [1,2,4,7].

Co-crystals have emerged in recent years as attractive, alternative solid forms of active pharmaceutical ingredients (APIs), with several studies aiming at the optimization of their physicochemical properties and/or biopharmaceutical performance [12]. They are made up of the API and of one or more co-formers, all of which are solids when pure and at ambient conditions, joined together in a stoichiometric ratio in a new crystalline structure [12,13]. The co-crystal components are associated in supramolecular synthons, most commonly linked by hydrogen bond interactions [12,13].

The supramolecular synthon approach, which looks for hydrogen bonding complementarities between the target molecule and the potential co-formers, is often used as a starting point for co-crystal screening [14,15]. In this context, it is interesting to investigate if DAP can be a guide for the screening of co-crystals of DHFR inhibitors containing this pharmacophore: the same type of supramolecular synthons is expected for DAP and for the DHFR inhibitors, although the molecular complexity of the latter may also play a role in the co-crystallization trial outcome. Although several studies can be found in literature concerning the investigation of co-crystals, and mainly of salts of TMP [16,17,18,19,20,21,22,23,24,25] and PMA [19,26,27,28,29,30,31,32,33,34] and, to a lesser extent, of DAP [35,36,37,38,39], to the best of our knowledge, an investigation comparing co-crystallization of these three related compounds with the same potential co-formers is not available.

The current work presents the results of an ongoing investigation of co-crystallization of trimethoprim, pyrimethamine and the core fragment 2,4-diaminopyrimide with different co-formers. Experimental results of co-crystallization screening, carried out in the same experimental conditions, are presented and discussed for DAP and TMP with two different types of co-formers that are capable of giving rise to different supramolecular heterosynthons, with the target molecules having, therefore, different competing/complementary effects towards the target homodimers: the three isomeric pyridinecarboxamides, picolinamide (PA), nicotinamide (NA) and isonicotinamide (INA), and the two xanthines, theophylline (THEO) and caffeine (CAF) (Figure 1). Some possible hydrogen-bonded motifs between the DAP scaffold and each of these types of co-formers are shown in Figure 1i and 1.j. The screening of co-crystals between PMA and the pyridinecarboxamides is also carried out, complementing our previous work [27] on the co-crystallization of this DHFR inhibitor with caffeine and theophylline.

## 2. Results and Discussion

COSMOquick software [40,41] was used in the search for the propensity of DAP, TMP and PMA to form co-crystals with the co-formers that are investigated in the current work. This approach takes into account the interaction between the target compound and the co-former as the excess enthalpy (Δ*H*_ex_) of the undercooled melt made up of both components in a specified proportion [41]. The software also calculates the empirical parameter *f*_fit_ for a 1:1 stoichiometry which, in addition to Δ*H*_ex_, also takes into account the flexibility of the molecules described by the number of rotatable bonds of the target molecule and the co-former [41]. Negative Δ*H*_ex_ values and low *f*_fit_ values should indicate a propensity for co-crystal formation.

Our previous results concerning PMA + THEO and PMA + CAF confirm successful (1:1) co-crystal formation for both systems, with polymorphism of the co-crystals being observed in both cases [27].

The results obtained for the systems that were investigated in the current work are presented in Table 1 and can provide a qualitative guide to the expectation of co-crystal formation. A general comparison of the estimated values for excess enthalpy of mixing indicate that a successful outcome is probable between DAP, TMP or PMA and the two investigated xanthines, although outcomes are less favorable for TMP. The estimated Δ*H*_ex_ values with the pyridinecarboxamide co-formers show a more favorable tendency for the co-crystallization of these three molecules with DAP and picolinamide with PMA. Other values are close to zero and are probably not meaningful, while indicating a smaller likelihood of the co-crystallization of TMP with the three pyridinecarboxamides.

### 2.1. (DAP/TMP/PMA) + Pyridinecarboxamides Binary Systems

Co-crystal formation of DAP, TMP and PMA with the isomeric pyridinecarboxamides was investigated experimentally using mechanochemistry as the screening method. Sample characterization was carried out using differential scanning calorimetry (DSC) together with the predicted (solid + liquid) binary phase diagrams, complemented by infrared spectroscopy (FTIR) or/and X-ray powder diffraction (XRPD) analysis.

Equation (1) was used to predict the liquidus for the binary systems made up of TMP or PMA and one of the pyridinecarboxamides and for DAP + PA and DAP + INA. Equations (1) and (2) [42] were used for DAP + NA system.

(1) $$\frac{1}{T_{fus}}=\frac{1}{T_{fus}^{*}}-\frac{R\ln x_{i}}{\Delta_{fus}H_{m}^{*}}$$

(2) $$\frac{1}{T_{fus}}=\frac{1}{T_{fus}^{cc}}-\frac{R}{\Delta_{fus}H_{m}^{cc}\;\;}\;\left\{\ln\left[x_{cc\;\;\;}{\left(1-x_{cc}\right)}^{3}\right]-\ln\left[x_{i}{\left(1-x_{i}\right)}^{3}\right]\right\}$$

In these equations, *T*_fus_ represents the liquidus temperature of a mixture of mole fraction *x*_i_. $T_{fus}^{*}$ and $\Delta_{fus}H_{m}^{*}$ are the melting temperature and molar melting enthalpy of the pure compound *i*; $T_{fus}^{cc}$ and $\Delta_{fus}H_{m}^{cc}$ are the corresponding values for the co-crystal. The melting temperature and enthalpy values presented in Table S1 (ESI) were used in the calculations. The experimental solidus was obtained from the onset of the first observed DSC peak and the liquidus from the corrected maximum of the second one [43,44].

For all the systems, except for DAP + NA, the binary (solid + liquid) phase diagrams (Figure S1; obtained from the DSC curves presented in Figures S2–S10) show only slight deviations from the predictions of a diagram with a simple eutectic, with the ideal liquid phase (eq. (1)) confirming that no co-crystals were obtained in the experimental conditions used. The infrared spectra shown as electronic supplementary material are, as expected, the sum of those of the target compound and of one of the polymorphs of the co-former (Figures S11 and S12—FTIR, DAP + PA and DAP + INA; Figures S13–S15—FTIR, TMP + PA, TMP + INA and TMP + NA; Figures S16–S18—FTIR and PMA + PA, PMA + INA, PMA + NA).

**Figure 2:**
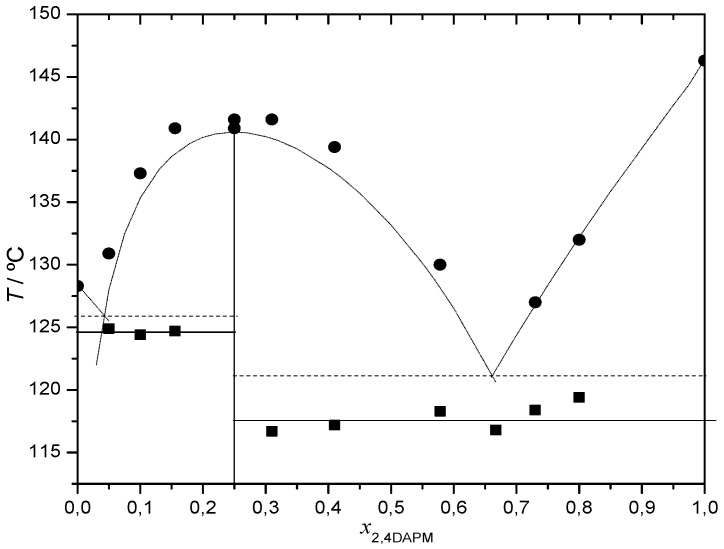
(Solid + liquid) binary phase diagram of DAP + NA; ■ and solid line—experimental solidus; ●—experimental liquidus. Dotted lines obtained using eqs. (**1**) and (**2**).

**Figure 3:**
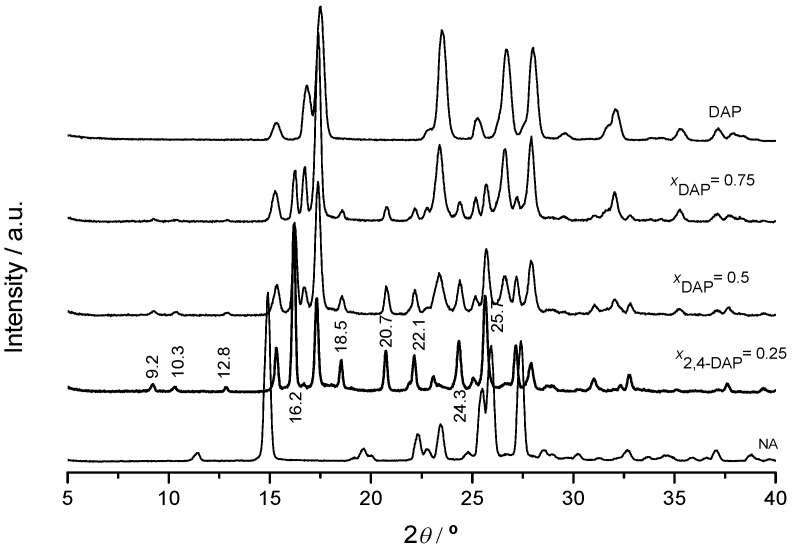
XRP diffractogram of DAP+NA mixtures of different compositions, obtained by LAG, ethanol-assisted.

A (1:3) DAP:NA co-crystal was synthesized by mechanochemistry, as confirmed by the experimental binary (solid + liquid) phase diagram shown in Figure 2, and by XRP (Figure 3) and FTIR (Figure S19). The binary (solid + liquid) phase diagram is quite well described by eq. (1) when an excess of DAP is present, and by eq. (2) in the vicinity of the co-crystal, between the two eutectic invariants at *T*_e1_ ≈ 124.5 °C, *x*_e1_ ≈ 0.04 and *T*_e2_ ≈ 117.5 °C, *x*_e2_ ≈ 0.65. Unique reflections are observed in the co-crystal X-ray powder diffractogram, for instance, at 2*θ* = 9.2, 10.3, 12.8, 16.2, 18.5, 20.7, 22.1, 24.3 and 25.7° (Figure 3). Shifts of the wavenumbers of N-H stretching modes are observed for the co-crystal relative to both pure NA and pure DAP, as well as of the C=O stretching mode of NA (Figure S19).

The pure co-crystal could also be obtained in a slurry of the pure components in ethanol, kept at 25 °C for 2 days (1:3 DAP + NA molar ratio).

#### General Remarks

The low propensity of DAP and both TMP and PMA to give rise to co-crystals with the isomeric pyridinecarboxamides, as predicted in the framework of COSMOquick, was confirmed. The DAP + NA exception is certainly a consequence of the greater simplicity of the DAP molecule, hence giving rise to fewer competing supramolecular association possibilities, and of the particular nicotinamide molecular structure. Concerning picolinamide, its lower co-crystal formation propensity when compared to their two isomers, with a different electronic structure in the ring, has been pointed out [45,46].

### 2.2. (DAP/TMP/PMA) + (THEO/CAF) Binary Systems

#### 2.2.1. 2,4-Diaminopyrimidine + theophylline and 2,4-Diaminopyrimidine + caffeine

**Figure 4:**
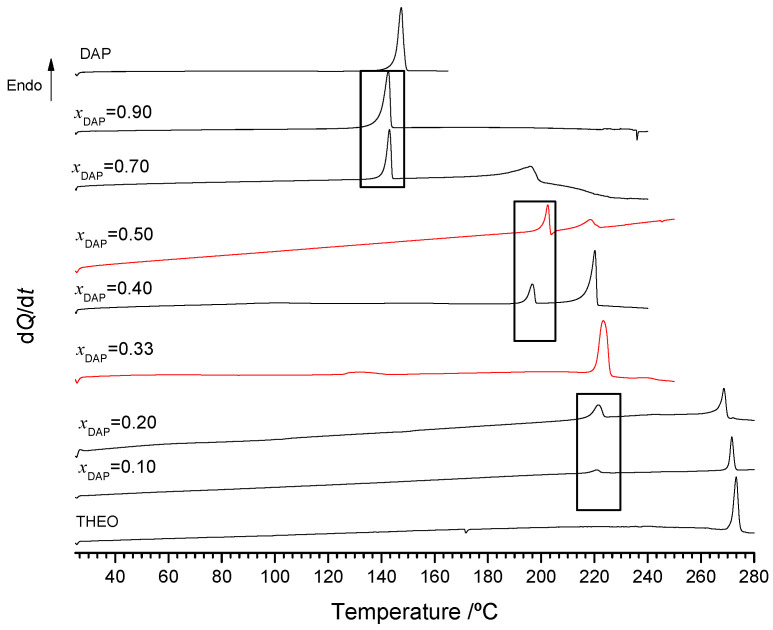
Representative DSC heating curves of DAP + THEO mixtures of different DAP mole fractions, *x*_DAP_, prepared by LAG, ethanol-assisted. *β* = 10 °C/min^−1.^

The representative DSC heating curves of binary mixtures of 2,4-diaminopirimidine + theophylline, prepared by ethanol-assisted grinding, are presented in Figure 4. A complex thermal behavior is observed with evidence of three invariant points, *T*_1_ = 137 °C, *T*_2_ = 192 °C and *T*_3_ = 218 °C, as indicated in the Figure. This behavior suggests that two co-crystals should be identified for this system.

**Figure 5:**
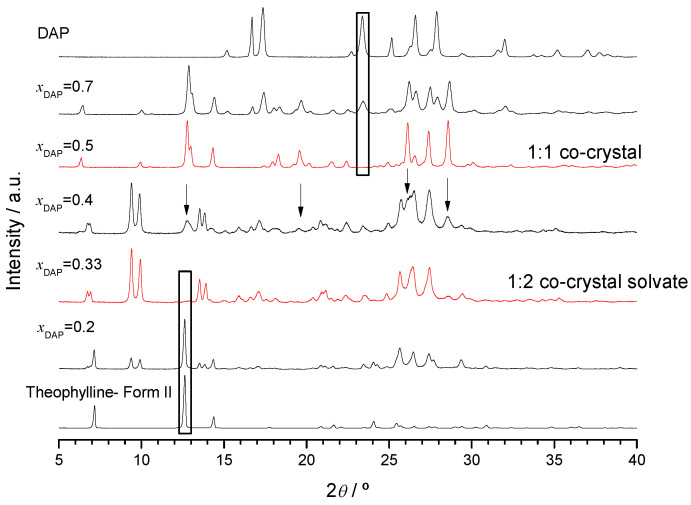
X-ray powder diffractograms of representative DAP + THEO mixtures prepared by ethanol-assisted grinding.

This is confirmed by X-ray powder diffractograms (Figure 5) that show unique patterns with no excess of either of the initial compounds for the (1:2) and (1:1) DAP:THEO mixtures (*x*_DAP_ = 0.33 and 0.50, respectively), indicating the formation of different multiple component solid forms with those stoichiometries. Unique reflections are observed for the (1:2) DAP:THEO solid at 2*θ* = 6.7, 6.9, 9.5, 9.9, 13.5, 13.9, 24.7 and 25.7°, for instance, and for the (1:1) multicomponent solid at 2*θ* = 6.4, 9.9, 13.0, 17.9, 18.3, 21.6, 26.1, 27.4 and 28.6°. For mixtures with *x*_DAP_ < 0.33, reflections due to theophylline are observed (besides those due to the (1:2) solid form); for *x*_DAP_ > 0.5, reflections of the (1:1) new solid form are seen and the excess of DAP is clearly identified. The X-ray powder diffractogram of the *x*_DAP_ = 0.4 mixture shows reflections of both (1:2) and (1:1) solid forms. The same conclusions are achieved by analysis of the FTIR spectra of representative mixtures shown in Figure S20.

**Figure 6:**
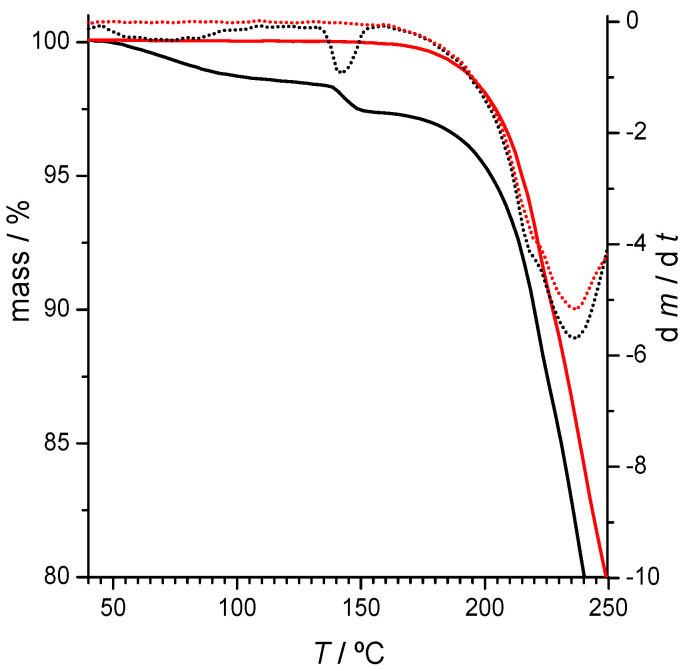
Thermogravimetric (solid lines) and derivative thermogravimetric (dotted lines) curves of *x*_DAP_ = 0.33 mixtures prepared by ethanol-assisted grinding (black lines) and by neat grinding (red lines), open pans.

Thermogravimetric analysis (Figure 6) indicates that the (1:2) co-crystal is a solvated one (see also Figures S21 and S22). The same X-ray powder diffractogram was obtained for a (1:2) DAP + THEO sample grinded with ethyl acetate assistance. The mass loss of about 1.5 % observed in the TG curve is compatible with a (1:2:0.5) DAP:THEO:H_2_O multicomponent solid.

**Figure 7:**
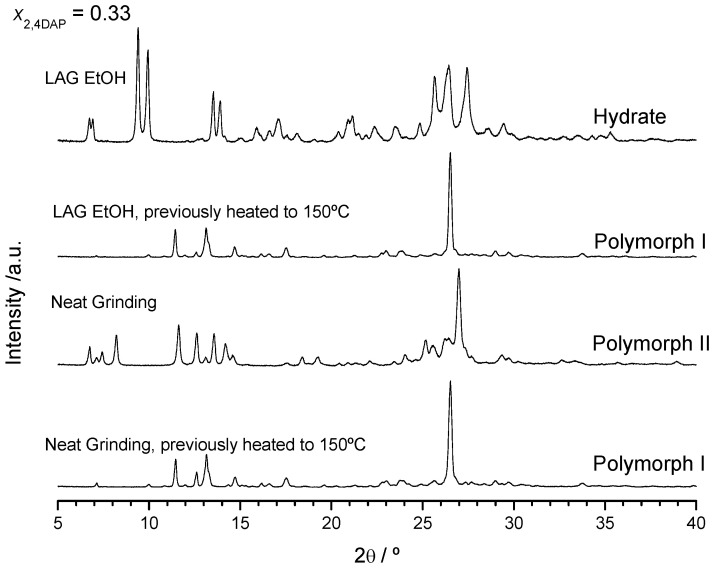
X-ray powder diffractograms of DAP + THEO mixtures, *x*_DAP_ = 0.33, obtained by different experimental methodologies.

The results obtained for x_DAP_ = 0.50 are the same when neat grinding is performed (Figure S23, ESI). However, neat grinding experiments carried out on (1:2) DAP + THEO mixtures give rise, as expected, to a solid form different from that obtained by LAG. This form is anhydrous (Figure 6) and has characteristic reflections in the X-ray powder diffractogram at 2*θ* = 7.4, 8.2, 13.6, 14.2, 25.1 and 27.0° (Figure 7). Interestingly, it gives rise, upon heating, to the same solid form obtained after the dehydration of the LAG-obtained co-crystal (Figure 7) with characteristic reflections at 2*θ* = 11.5, 13.1, 14.7, 17.5 and 26.5°. DSC curves are shown in Figure S21.

**Figure 8:**
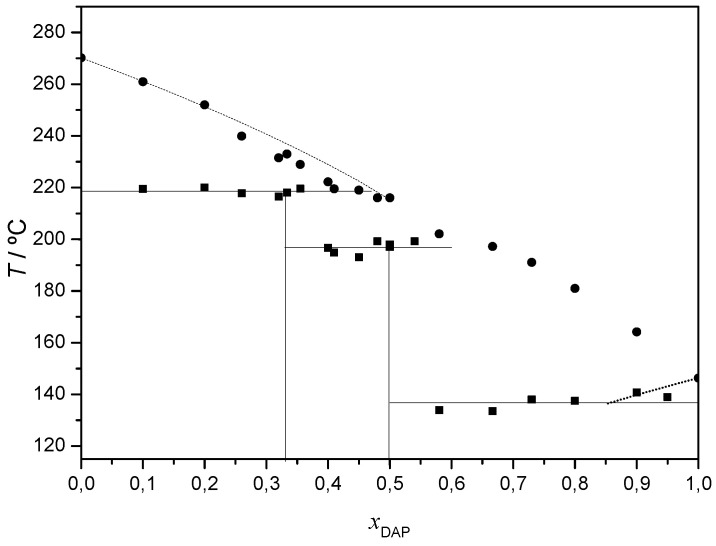
(Solid + liquid) binary phase diagram of DAP + THEO: ■ and solid horizontal line—experimental solidus; ●—experimental liquidus; vertical line—co-crystal composition. Dotted lines obtained using eq. (1).

A tentative (solid + liquid) binary phase diagram presented in Figure 8 may be proposed where two incongruently melting co-crystals are identified at (1:2) and (1:1) DAP:THEO.

In conclusion, for the DAP + THEO system, a (1:1) co-crystal was identified, as well as two polymorphic (1:2) co-crystals and a (1:2) DAP:THEO co-crystal hydrate.

Concerning DAP + CAF, in the experimental conditions used in the current work, co-crystal formation was not successful. This is evident from the binary (solid + liquid) phase diagram (Figure S24; representative DSC curves shown in Figure S25) and from FTIR spectra (Figure S26).

#### 2.2.2. Trimethoprim + theophylline and Trimethoprim + caffeine

**Figure 9:**
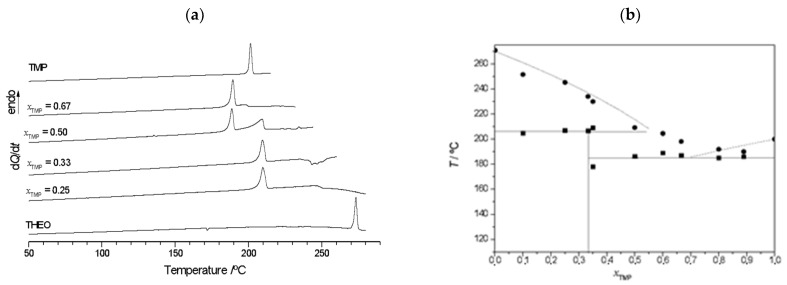
(**a**) Representative DSC heating curves of TMP + THEO mixtures of different TMP mole fractions, *x*_TMP_, prepared by LAG, ethanol-assisted. *β* = 10 °C/min^−1^. (**b**) (Solid + liquid) binary phase diagram of TMP + THEO ■ and solid horizontal line—experimental solidus; ●—experimental liquidus; vertical line—co-crystal composition. Dotted lines obtained using eq. (1).

**Figure 10:**
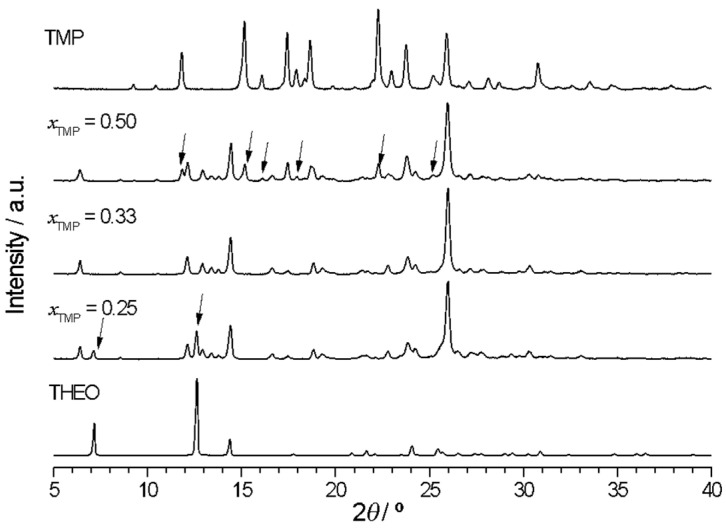
X-ray powder diffractogram of TMP + THEO mixtures of different compositions, obtained by LAG, ethanol-assisted.

The DSC curves shown in Figure 9a and the proposed (solid + liquid) binary phase diagram (Figure 9b) point to (1:2) TMP:THEO co-crystal formation, which is confirmed by X-ray diffraction and FTIR investigation (Figure 10 and Figure S27, respectively). In these Figures, the excesses of the pure components are indicated by arrows.

**Figure 11:**
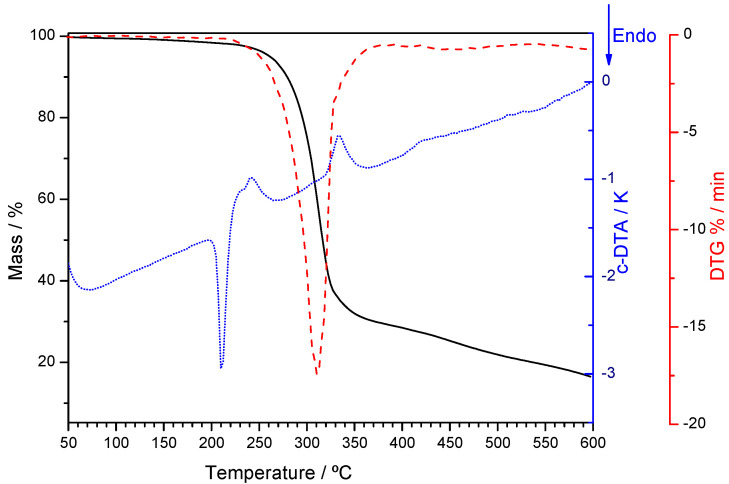
Thermogravimetric curve (solid black line), derivative thermogravimetric curve (dashed red line) and DTA heating curve (dotted blue line) of (1:2) TMP:THEO co-crystal.

The thermogravimetric curve of the co-crystal (Figure 11) shows only a residual mass loss.

**Figure 12:**
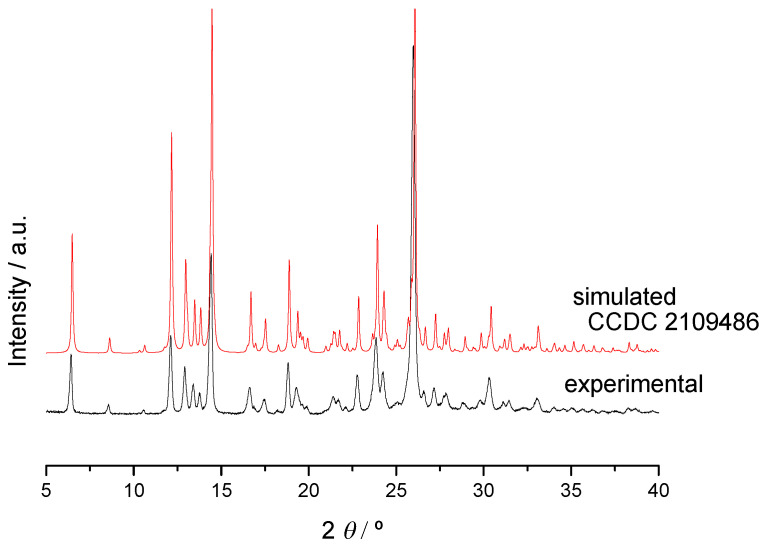
Experimental X-ray powder diffractogram of (1:2) DAP + THEO 1:2 co-crystal and simulated for the (1:2) + 17 % H_2_O solved structure, CCDC 2109486.

The crystalline structure of a (1:2) TMP:THEO (17% H_2_O) was solved (CCDC 2109486) as described in Section 3.7 and discussed in Section 2.2.3. The simulated X-ray powder diffractogram for this co-crystal matches the experimental one of the co-crystal obtained by grinding (Figure 12).

In the experimental conditions used in the current work, as it was also observed for DAP + CAF, co-crystal formation between trimethoprim and caffeine was not successful (see Figures S28–S30).

#### 2.2.3. Crystalline Structures and Hirshfeld Surfaces Analysis

A comparison of the propensity for co-crystal formation with a common set of co-formers was performed in the preceding sections for the DAP scaffold and the derived dihydrofolate reductase inhibitors, trimethoprim and pyrimethamine. A comparison of the intermolecular association in crystals of DAP, TMP and PMA with the same set of co-formers is, naturally, also of interest.

Several attempts, using different experimental approaches, were tried in order to obtain single crystals of the multicomponent solids identified in this work that are suitable for crystal structure resolution. This is a hard task, with unpredictable successful results. Single crystals of the (1:2) TMP:THEO co-crystal could be obtained, as described in Section 3.7, and its crystalline structure solved. Additionally, when searching for DAP:THEO co-crystals, single crystals of a new (1:2:1) DAP:THEO:EtAc solvate were obtained. Crystallographic data for both structures are presented in Section 3.7. A comparison of the intermolecular association in these two new crystalline structures, with the same co-former, is presented in this section. Comparison is also made with the (1:1) PMA:THEO co-crystal structure solved by Delori et al. [28].

**Figure 13:**
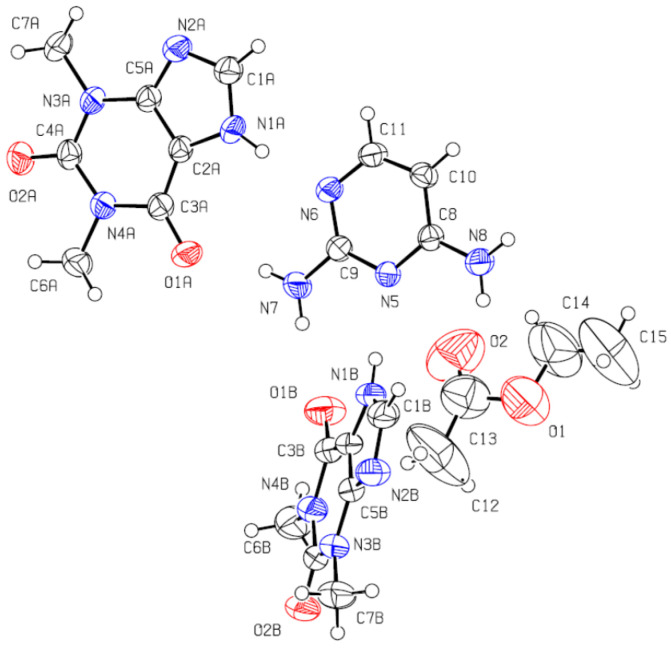
ORTEP diagram of the (1:2:1) 2,4-diaminopyrimidine:theophylline:ethyl acetate, CCDC 2108308. Ellipsoids are drawn at the 50% probability level. Hydrogen atoms are drawn as spheres of arbitrary radius.

**Figure 14:**
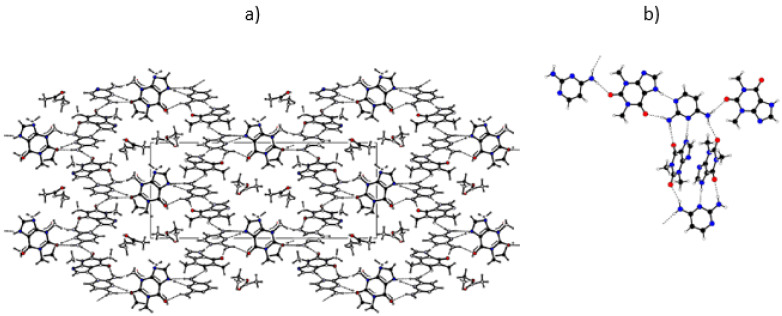
(**a**) Packing diagram for (1:2:1) DAP:THEO:EtAc viewed along the a axis. (**b**) Detail of the hydrogen bonded ribbons that join the 2,4-diaminopyrimidine and the theophylline molecules.

The ORTEP diagram, with the numbering scheme for (1:2:1) DAP:THEO:EtAc solvate, is shown in Figure 13. The unit cell and illustrative images of the crystalline arrangement and of the intermolecular hydrogen bonds are shown in Figure 14 and in Table 2.

The molecules crystallize in a monoclinic crystal structure, under the common P2_1_/c space group. The 2,4-diaminopyrimidine and the theophylline molecules are joined together in ribbons, with each 2,4-diaminopyrimidine molecule H-bonded to four theophylline molecules (Figure 14b and Table 2). The planar ribbons containing the DAP molecules are interconnected by skewed THEO molecules (an angle of 47.1° is observed between the DAP ring plane and that of these theophylline molecules). The ribbons run along the (100) direction, forming channels that are occupied by the ethyl acetate solvent molecules. Concerning the solvent molecules, very weak contacts [47], only with theophylline molecules, are observed (O2···H1AC1A: O2···H1A = 2.457 Å; O2···C1A = 3.388 Å, O2···H1A–C1A = 179.5°; C14H14B···N2A: H14B···N2A = 2.625 Å; C14···N2A = 3.461 Å, C14–H···N2 = 145.7°; C15H15A···O1B: H15A···O1B = 2.713 Å; C15···O1B = 3.492 Å, C15–H15A···O1B = 138.6). There are no intermolecular hydrogen bonds between DAP molecules.

**Figure 15:**
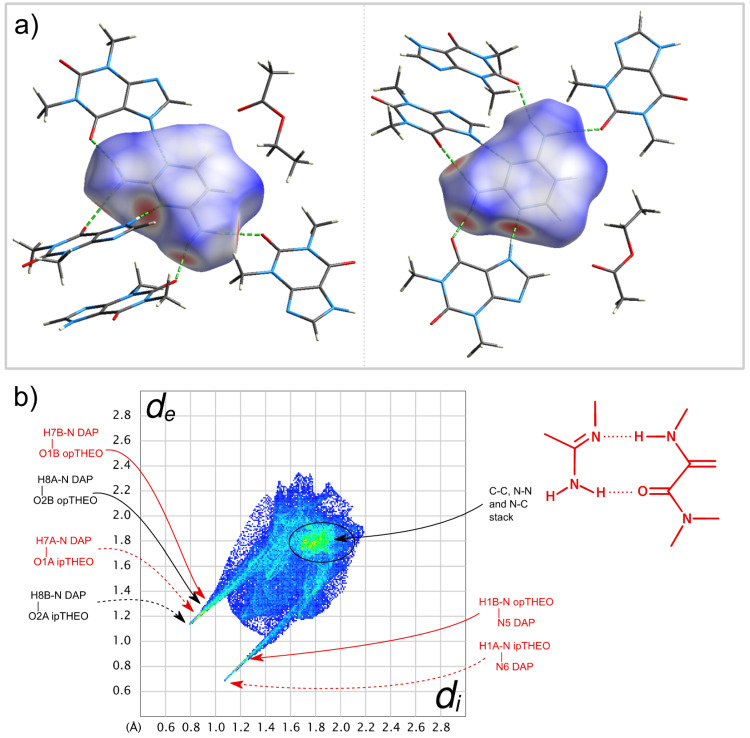
(**a**) Two views of the close contacts of the semitransparent *d*_norm_-mapped Hirshfeld surface of DAP molecule. (**b**) Hirshfeld fingerprint plot of the DAP molecule in the (1:2:1) DAP:THEO:EtAc co-crystal solvate. Some important pairs of contacting atoms are identified and assigned to fingerprint plot features, with dashed arrows for “in-plane” (ipTHEO) and solid arrows for “out-of-plane” (opTHEO) THEO molecules. Contacts within the $R_{2}^{2}\left(9\right)$ synthon between the DAP scaffold and THEO, shown at the side, are colored in red. *d*_i_—the distance (Å) from the Hirschfeld surface to the nearest atom center in its interior; *d*_e_—distance (Å) from the Hirschfeld surface to the nearest atom center in its exterior.

The close contacts involving the DAP molecule in the co-crystal are highlighted in its Hirshfeld surface, shown in Figure 15a, and consist of six H-bonds. In four of these, the DAP NH_2_ groups act as donors to different THEO O=C groups, and in the other two, each N atom in the ring acts as acceptor from the NH groups of two THEO molecules.

The four NH···O interactions are expressed in the left spike of the fingerprint plot in Figure 15b. The tip of the spike corresponds to the stronger H bonds, towards the approximately co-planar THEO molecules in the above-mentioned ribbons. The two N···HN contacts, identified as the right spike in the plot, follow the same trend, with stronger H bonding involving the ribbon THEO molecules. It is worth mentioning the green area close to *d*_i_ ≈ *d*_e_ = 1.8 Å correspond to heavy atom contacts due to DAP···THEO π-π stacking interactions [48].

**Figure 16:**
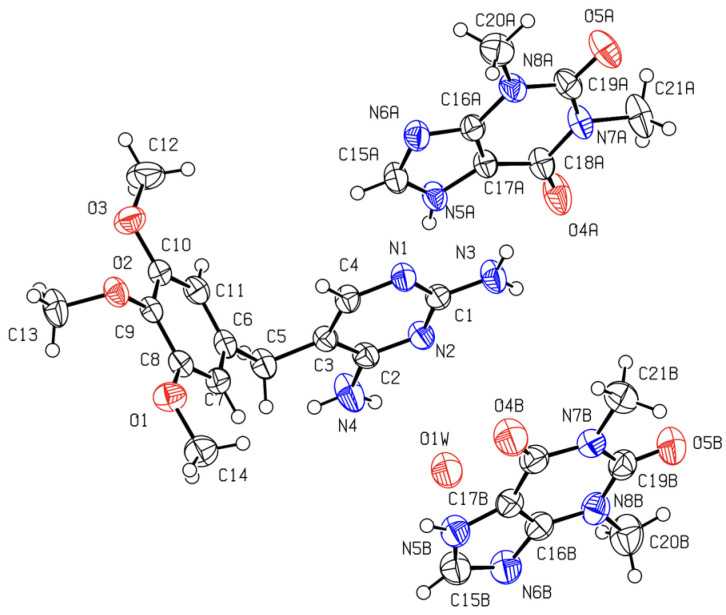
ORTEP diagram of the (1:2) trimethoprim:theophylline, CCDC 2109486. Ellipsoids are drawn at the 50% probability level. Hydrogen atoms are drawn as spheres of arbitrary radius.

**Figure 17:**
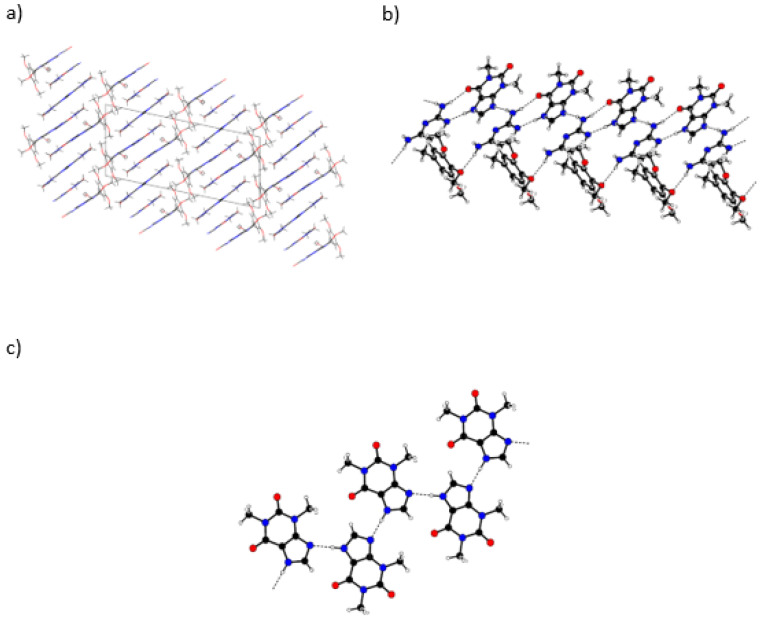
(1:2) trimethoprim:theophylline, CCDC 2109486: (**a**) Unit cell packing viewed along the b axis; (**b**) hydrogen bonded chains joining trimethoprim and theophylline molecules; (**c**) hydrogen bonded chains joining the second theophylline molecule.

Single crystals of a (1:2) trimethoprim theophylline (partially solvated) co-crystal were obtained as described in Section 3.7. The ORTEP diagram, with the numbering scheme, is shown in Figure 16 and in the unit cell, and illustrative images of the crystalline arrangement and of the intermolecular hydrogen bonds are shown in Figure 17 and in Table 3.

The molecules of trimethoprim and theophylline are joined in chains by H-bonds. One of the chains is assembled by the trimethoprim and one of the two theophylline independent molecules (Figure 17b). The other theophylline independent molecules form a different chain (Figure 17c). In this planar chain, the theophylline molecules are joined by N5B–H5B∙∙∙N6B hydrogen bonds, as in pure theophylline polymorph II, CCDC128707 [49], although, in pure THEO, the molecules are not in the same plane (Figure S31). The chains pack parallel to each other (see Figure 17a; structure viewed along the b axis). An oxygen atom (water molecule) is located close to one of the amine groups of trimethoprim, but with just a 17% occupation, accounting for a weak solvation. It is possible that the 17% of occupation found in the refinement of the data coming from a single data collection is just a “snapshot” of a continuous series of possible solvent/compound ratios. There is evidence that this non-stoichiometric solvate can desolvate to the two-component phase without changing the main structural characteristics, since powder diffractograms collected before and after forced hydration do not change (Figure S32). It is even possible that the water molecules escape the lattice at room temperature due to their small size and weak interactions within the crystal main structure.

There is only a small change in trimethoprim conformation in the co-crystal with an angle of 80° between the planes of the two rings, slightly greater than in pure TMP crystals, 70° (CCDC607118 [50]). In the co-crystal, as in pure trimethoprim [50], the oxygen (O2) of the middle methoxy group is hydrogen-bonded to a NH group of a different TMP molecule, N4H4 in the co-crystal and N3H3 in pure TMP. As in the (1:2:1) DAP:THEO:EtAc structure, no NH···N hydrogen bonds involving the aminopyrimidine scaffold are observed between TMP molecules.

**Figure 18:**
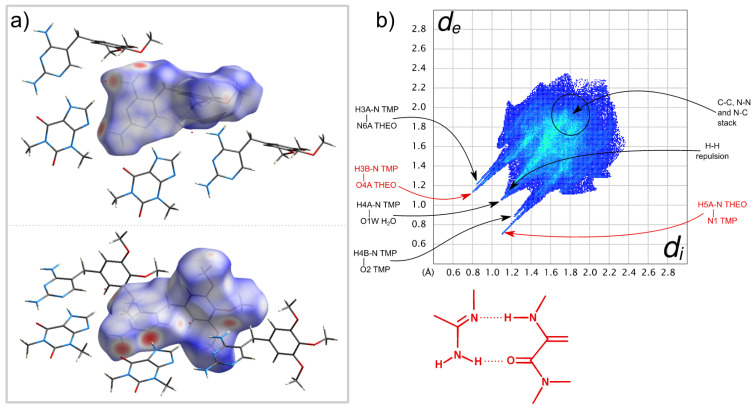
(**a**) Two views of the semitransparent *d*_norm_-mapped Hirshfeld surface of a TMP molecule; (**b**) Hirshfeld fingerprint plot of the TMP molecule in the (1:2) TMP:THEO (17% H_2_O) co-crystal. Some important pairs of contacting atoms are identified and assigned to fingerprint plot features. Contacts within the $R_{2}^{2}\left(9\right)$ synthon between the DAP scaffold and THEO, shown at the bottom, are colored in red. *d*_i_—the distance (Å) from the Hirschfeld surface to the nearest atom center in its interior; *d*_e_—distance (Å) from the Hirschfeld surface to the nearest atom center in its exterior.

The Hirshfeld surface of the TMP molecule in TMP:THEO, presented in Figure 18a and in the fingerprint plot (Figure 18b) show similar features to DAP in the previously mentioned co-crystal. The evidence of different donating and accepting H bonds are manifested in the complex structure of their two corresponding spikes. In both co-crystals, the NH···N type between the NH group of THEO and one of the ring N atoms of the diaminopyrimidine scaffold is the strongest interaction—(*d*_i_,*d*_e_)/Å ≈ (1.1,0.7)—despite carbonyl generally being a better acceptor of H bonding than aromatic ring nitrogen. It is worth mentioning that in both of the co-crystal structures solved in this work, the synthon that is specially highlighted in Figure 15 and Figure 18 plays a major role. This synthon is also observed in the (1:1) PMA:THEO co-crystal structure solved by Delori et al. [28]. In this latter co-crystal, of a different stoichiometry, PMA homodimers are observed. The presence of π-π stacking is noticed in both Hirshfeld surfaces around *d*_i_ ≈ *d*_e_ = 1.8 Å.

#### 2.2.4. General Remarks

The co-crystal screening resulted in the successful synthesis of multicomponent solid forms of all three target molecules with theophylline. DAP co-crystals revealed a greater variety of stoichiometries (1:1, 1:2 DAP:THEO) and even polymorphism in the case of the 1:2 stoichiometry. Likewise, theophylline was a viable co-former with TMP, however with a single form of 1:2 TMP:THEO stoichiometry. This concurs with previous findings, where two co-crystal polymorphs were obtained for (1:1) PMA:THEO [27,28].

The crystalline structure of the (1:2) TMP:THEO was resolved in this work, as well as an ethyl acetate solvate of DAP:THEO in the same stoichiometry. Both structures lack hydrogen bonding between the aminopyrimidine scaffolds, and both share the common synthon described above in Figure 1j. and previously recognized in PMA:THEO [28]. This synthon, a $R_{2}^{2}\left(9\right)$ hydrogen bonding motif, identified in Figure 15 and Figure 18, involves the NH group of theophylline as a hydrogen bonding donor. Contrary to theophylline, caffeine has no hydrogen-bonding donor groups, which could be a possible reason for its weaker co-former performance with the target compounds. It could not form co-crystals with either DAP or TMP, despite the previously reported successful synthesis of a polymorphic (1:1) co-crystal with PMA [27]. In both of these latter PMA co-crystals, the hydrogen bonds among pyrimethamine molecules involve all 2,4-diaminopyrimidine donor and acceptor groups in a ribbon pattern that is similar to that observed in pyrimethamine polymorph I [51].

## 3. Experimental Procedures

### 3.1. Materials

2,4-Diaminopyrimidine was supplied by TCI Europe, *x* > 0.98 and confirmed by XRPD to be the solid form described by Hützler et al. [37] (CCDC 1499993). Pyrimethamine was from Aldrich (St. Louis, MO, USA) (form I [27], CCDC 193733 [51]), specified chemical purity *x* = 0.9899, and trimethoprim from Sigma Aldrich (St. Louis, MO, USA), *x* > 0.98, crystal structure CCDC 607118, solved by Rauf et al. [52]. Nicotinamide (Form I, [53] picolinamide (polymorph II, [54,55]), isonicotinamide (polymorph I, reference code EHOWIH01 [56]) and theophylline, (Form II CCDC 128707 [49], were all from Aldrich (St. Louis, MO, USA) with specified purities, namely, *x* = 0.995, 0.98, 0.99 and 0.99, respectively. Caffeine, *x* ≥ 0.99, form II CCDC 610381 [57], was acquired from Fluka (Charlotte, NC, USA). Ethanol and ethyl acetate (EtAc), both *x* = 0.998, were from Fischer (Hampton, NH, USA). Poly(ethylene oxide) (PEO), MW = 600,000, from Sigma (St. Louis, MO, US), was used in gel crystallization experiments.

### 3.2. Co-Crystals Screening

A preliminary co-crystal in silico screening was carried out using the BIOVIA COSMOquick 2020 software (Vélizy-Villacoublay, France) [40,41]. COSMOquick relies on the COSMOfrag fragmentation approach which employs a database of previously computed σ-profiles [58] for a set of about 190,000 compounds and its estimation of thermodynamic properties, avoiding the use of costly quantum mechanical calculations, to implement its cocrystal screening capability. The propensity for cocrystal formation is estimated from the excess enthalpy of the virtually subcooled liquid mixture of the components. In order to account for the negative effect of conformational flexibility in cocrystal formation, a partial empirical function based on the number of rotatable torsions is introduced that punishes highly flexible compounds in a screening.

Mechanochemistry was chosen for the experimental co-crystals screening. A MM400 Retsch ball mill, with 10 mL stainless steel jars and two 7 mm diameter stainless balls per jar, was used. Typically, a total mass of ~70 mg was used, and grinding was performed neat or with the assistance of 10 μL ethanol, at 15 or 30 Hz, for times ranging from 30 to 90 min.

### 3.3. Differential Scanning Calorimetry (DSC)

DSC experiments were carried out using a Perkin Elmer DSC7 (Norwalk, CT, USA) calorimeter, with an intracooler unit at -10 °C (ethylene glycol-water 1:1 *v/v* cooling mixture), and a 20 mL/min^-1^ nitrogen purge. Aluminum pans suitable for volatile substances (Perkin Elmer, 30 μL) were employed, with an empty pan as reference. Temperature and enthalpy calibrations were performed with certified reference materials, following the procedure described elsewhere [59]. Heating rates of 10 °C/min^-1^ were used. The DSC curves were analyzed with Perkin Elmer Pyris software, version 3.5.

### 3.4. Thermogravimetric Analysis (TGA)

A Netzsch TG 209 F3 (Selb, Germany) Tarsus equipment was employed using alumina pans. Sample masses *m* ≈ 10 mg were used, and temperature was scanned from 30 to 600 °C in a N_2_ atmosphere (25 mL/min) at a *β* = 10 °C/min scanning rate. Temperature calibration was performed using indium, tin, bismuth, zinc, aluminum and silver standards. Mass and linearity of the balance was verified with standard masses of the order of milligrams. Data was analyzed by Netzsch Proteus Thermal Analysis, version 7.2.0. software.

### 3.5. Infrared Spectroscopy (FTIR)

Infrared spectra were obtained in a ThermoNicolet 380 (Thermo Scientific TM, CA, USA) Fourier transform infrared spectrometer, with a Smart Orbit Diamond Attenuated Total Reflection (ATR) system, 64 scans and a 2 cm^−1^ spectral resolution.

### 3.6. X-ray Powder Diffraction (XRPD)

Powder diffractograms were obtained using a Rigaku MiniFlex 600 (Tokyo, Japan) diffractometer, Cu Kα radiation (λ = 1.540598 Å), with a D/teX-Ultra high-speed detector. Calibration was performed using silicon as an external calibrant. A scan range from 3° to 40° 2*θ* was used.

### 3.7. Single-Crystal X-ray Diffraction (SXD)

Single crystals of a (1:2:1) 2,4-diaminopyrimide:theophylline:ethyl acetate solvate were obtained from evaporation, at room temperature, of an ethyl acetate solution prepared by dissolving a (2.5:3) DAP:THEO sample and obtained by grinding.

Single crystals of a (1:2) trimethoprim:theophylline co-crystal were obtained by gel crystallization: 13.0 mg of the co-crystal was obtained by grinding 2 mL ethanol + water 1:1 *v/v*, 0.08 g/L poly(ethylene oxide), following the procedure described by Choquesillo-Lazarte and Garcia-Ruiz [60].

In the determination of crystal structures of monocrystals by X-ray diffraction analysis, a Bruker-Nonius Kappa Apex II (Bruker, Karlsruhe, DE) CCD diffractometer was used, using MoKα radiation (λ = 0.71073 Å). Structures were solved by direct methods and by conventional Fourier Synthesis (SHELXS-97) [61]. The refinement of the structures was performed by full-matrix least-squares on F^2^ (SHELXL-97) [62]. Hydrogen atoms attached to carbon atoms were placed at calculated positions and refined as riding. Hydrogen atoms attached to nitrogen atoms were located in a difference Fourier synthesis and their coordinates were freely refined. For the (1:2) TMP:THEO co-crystal, at the final refinement stages, a peak in a difference Fourier map of 1.54 e/Å^3^ persisted and an oxygen atom was tentatively added to the model, refining to near 17% occupancy that may correspond to a water molecule whose hydrogen atoms were not located.

A summary of the data collection and refinement details is given in Table 4.

### 3.8. Hirshfeld Surfaces Analysis

The CIF files were used as starting points to generate the Hirshfeld surfaces and fingerprints [63,64] using CrystalExplorer, version 17.5. (University of Western Australia), which was also used for their analysis. CrystalExplorer normalizes the C-H and N-H bonds as those obtained from neutron diffraction experiments (C-H 1.083 Å, N-H 1.009 Å). The surfaces were generated using a “very high” resolution.

## 4. Conclusions

As a result of this work, new co-crystals were discovered and characterized by DSC, FTIR and XRPD: (1:3) DAP:NA, (1:1) DAP:THEO, two polymorphic (1:2) DAP:THEO cocrystals, a (1:2:1) DAP:THEO:EtAc solvate and a (1:2) TMP:THEO co-crystal; the crystalline structures of the latter two were solved.

In Table 5, a summary of the results of the co-crystal screening outcomes is presented. This work demonstrated the possible use of the pharmacophore of dihydrofolate reductase inhibitors as a guide for co-crystal screening, recognizing some similar trends in the outcome of association in the solid state, and in the molecular aggregation in the co-crystals characterized by the same supramolecular synthons. Nevertheless, the co-crystal outcome for the API will always be more or less influenced by the remaining molecular fragments and the result of a balance of all interactions in the crystal lattice environment.

The comparison of the experimental co-crystallization outcome with the prediction of the virtual co-crystal screening with COSMOquick confirms the low propensity of DAP, TMP and PMA to co-crystallize with pyridinecarboxamides, with a successful outcome only observed for DAP + nicotinamide. Co-crystallization attempts with theophylline were well predicted; while using caffeine as co-former, they were not successful for DAP and TMP, contrarily to what was predicted by the in silico method. This shows that the method is useful as a preliminary guide to co-crystal screening, although it is limited by misrepresentation of complete conformational space, explicit hydrogen bonding, crystal packing and kinetic factors.

## Figures and Tables

**Table 1:** COSMOquick parameters, Δ*H*_ex_ (kcal mol^−1^) and *f*_fit_, obtained for co-crystal screening between the target compounds 2,4-diaminopyrimidine, trimethoprim and pyrimethamine, and the co-formers, picolinamide, isonicotinamide, nicotinamide, theophylline and caffeine; *x* is the target compound mole fraction.

	*f*_fit_ *x* = 0.50	Δ*H*_ex_ *x* = 0.33	Δ*H*_ex_ *x* = 0.50	Δ*H*_ex_ *x* = 0.67	*f*_fit_ *x* = 0.50	Δ*H*_ex_ *x* = 0.33	Δ*H*_ex_ *x* = 0.5	Δ*H*_ex_ *x* = 0.67	*f*_fit_ *x* = 0.50	Δ*H*_ex_ *x* = 0.33	Δ*H*_ex_ *x* = 0.5	Δ*H*_ex_ *x* = 0.67
	Picolinamide				Isonicotinamide				Nicotinamide			
DAP	1.9	−0.14	−0.15	−0.12	1.9	−0.15	−0.16	−0.14	1.9	−0.13	−0.14	−0.12
TMP	4.6	0.02	0.02	0.02	4.6	0.04	0.04	0.03	4.6	0.05	0.04	0.03
PMA	3.0	−0.14	−0.15	−0.12	3.0	−0.06	−0.06	−0.05	3.0	−0.04	−0.04	−0.03
	Theophylline				Caffeine							
DAP	1.1	−0.46	−0.54	−0.49	1.0	−0.50	−0.62	−0.60				
TMP	3.9	−0.26	−0.28	−0.25	4.0	−0.14	−0.16	−0.14				
PMA	2.2	−0.44	−0.50	−0.44	2.0	−0.60	−0.70	−0.60				

**Table 2:** Hydrogen bonds in (1:2:1) DAP:THEO:EtAc solvate.

D–H···A	D–H/Å	H···A/Å	D···A/Å	D–H···A/°
(1:2:1) DAP:THEO:EtAc				
N1A–H1A···N6	0.86	1.91	2.760(3)	171
N1B–H1B···N5	0.86	2.05	2.907(3)	178
N7–H7A···O1A	0.86	2.11	2.957(3)	167
N7–H7B···O1B	0.86	2.20	2.992(3)	152
N8–H8A···O2B^a^	0.86	2.16	2.970(3)	156
N8–H8B···O2A^b^	0.86	2.08	2.932(3)	174
*a:* −1 − *x*, −*y*, 1 − *z*; *b*: 2 *+ x*, 1/2 − *y*, 1/2 *+ z*				

**Table 3:** Geometric parameters of hydrogen bonds for (1:2) trimethoprim:theophylline. (*D* and *A* are the donor and the acceptor atoms, respectively.)

D–H···A	D–H/Å	H···A/Å	D∙∙∙A/Å	D–H∙∙∙A/°
N3–H3A∙∙∙N6A*^i^*	0.86	2.21	3.064(2)	171
N3–H3B∙∙∙O4A	0.86	2.09	2.933(2)	166
N4–H4B∙∙∙O2*^i^*	0.86	2.23	2.927(2)	139
N5A–H5A∙∙∙N1	0.86	1.97	2.820(2)	170
N5B–H5B∙∙∙N6B^v^	0.86	1.90	2.762(2)	175
*i*: *x*, −1 *+ y*, *z*; *ii*: −*x*, 1/2 *+ y*, 1/2 − *z.*				

**Table 4:** Crystal data and structure refinement parameters of the (1:2:1) DAP:THEO:EtAc and (1:2) TMP:THEO (0.17 H_2_O) co-crystals.

	(1:2:1) DAP:THEO:EtAc CCDC2108308	(1:2) TMP:THEO (0.17 H_2_O) CCDC2109486
Temperature/K	293	293
Empirical formula	C_22_ H_30_ N_12_ O_6_	C_28_H_34_N_12_O_7.17_
Formula weight	558.58	653.46
Wavelength/Å	0.71073	0.71073
Crystal system	Monoclinic	Monoclinic
Space group	P2_1_/c	P2_1_/c
*a/*Å	7.4376(10)	12.5845(6)
*b*/Å	29.309(4)	9.0152(4)
*c*/Å	13.4200(14)	27.8422(13)
*β*/º	112.968(6)	101.9030(10)
Volume/Å^3^	2693.5(6)	3090.8(2)
Z	4	4
Calculated density/(g/cm^3^)	1.377	1.404
Absorption coefficient/mm^–1^	0.104	0.105
F(000)	1176	1374
θ range for data collection/deg.	2.15–25.49	1.49–25.72
Index ranges	–8 < h < 9,–35 < k < 35, –16 < l < 16	–15 < h < 15,–10 < k < 10,–33 < l < 33
Reflections collected/unique	28210/4963	57612/5861
Completeness to θ_max_	99.9%	99.6 %
Data/restraints/parameters	4963/0/367	5861/0/454 [*R*(int) = 0.0293]
Goodness-of-fit on F^2^	1.030	1.025
Final R index [I > 2σ(I)]	*R1* = 0.0599 *wR2* = 0.1428	*R1* = 0.0388 *wR2* = 0.1052
R index (all data)	*R1* = 0.1140 *wR2* = 0.1744	*R1* = 0.0484 *wR2* = 0.1144
Largest diff. peak and hole (e Å ^–3^)	–0.296 and 0.488	–0.175 and 0.252

**Table 5:** Summary of co-crystal screening results for DAP, TMP and PMA, with the co-formers PA, INA, NA, THEO and CAF.

Co-Former	Target		
	DAP	TMP	PMA
PA	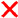	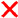	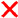
INA	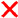	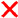	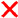
NA	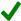	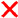	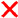
THEO	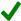	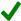	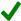 (27,28)
CAF	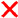	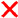	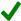 (27)

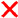
—no co-crystal; 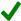
—co-crystal formed.

## Data Availability

Data are available from the corresponding author upon reasonable request.

## Supplementary Materials

Figure S1: (Solid + liquid) binary phase diagrams of DAP + PA/INA; TMP + PA/INA/NA; PMA + PA/INA/NA; Figures S2–S10: DSC heating curves for binary mixtures DAP + PA/INA/NA, TMP + PA/INA/NA, PMA + PA/INA/NA; Figures S11–S18: FTIR of binary mixtures DAP + PA/INA, TMP + PA/INA/NA, PMA + PA/INA/NA; Figure S19: FTIR of binary mixtures DAP + NA; Figure S20: FTIR of DAP + THEO mixtures; Figure S21: TG curves of DAP + THEO, xDAP = 0.33 NG, open and pierced pans; Figure S22: DSC heating curves of xDAP = 0.33, LAG and NG, submitted to different thermal treatments; Figure S23; XRPD of (1:1) DAP:THEO prepared by LAG and by NG; Figure S24: (Solid + liquid) binary phase diagram of DAP + CAF; FigureS25 and S26: DSC heating curves and FTIR of binary mixtures DAP + CAF; Figure S27: FTIR of binary mixtures TMP + THEO; Figure S28: (Solid + liquid) binary phase diagram of TMP + CAF; Figures S29 and S30: DSC heating curves and FTIR of binary mixtures TMP + CAF; Figure S31: Representation of hydrogen-bonded chains in theophylline, polymorph II; Figure S32: Comparison of XRPD LAG (1:2) TMP:THEO co-crystal, LAG co-crystal exposed to a saturated water atmosphere for 15 days and simulated from CCDC2109486; Table S1: melting temperature and enthalpy of the compounds used in the current work. CIF files with the single crystal X-ray diffraction structures of (1:2)TMP:THEO (17% H2O), CCDC 2109486, and (1:2:1) DAP:THEO:EtAc, CCDC 2108308, are available from the Cambridge Crystallographic Data Center.

## Sample Availability

Samples of the compounds are not available from the authors.
