# Supplementary material for: Dihydrofolate Reductase Inhibitors: The Pharmacophore as a Guide for Co-Crystal Screening
Source: Molecules. 2021 Nov 6;26(21):6721. doi: 10.3390/molecules26216721 (PMC8587188; doi:10.3390/molecules26216721)
Supplement: Supplementary file 1 [file molecules-26-06721-s001.zip › molecules-1429128-SI.pdf]

molecules

Supplementary Information

Dihydrofolate Reductase Inhibitors: The Pharmacophore as a Guide for Co-Crystal Screening

João A. Baptista [1], Mário T.S. Rosado [1], Ricardo A.E. Castro [1,2], António O.L. Évora [1,3], Teresa M.R. Maria [1], Manuela Ramos Silva [4], João Canotilho [1,2] and M. Ermelinda S. Eusébio [1,*]

[1] CQC, Departamento de Química, Universidade de Coimbra,3004-535 Coimbra, Portugal; jabaptista@qui.uc.pt (J.A.B.); mario.rosado@qui.uc.pt (M.T.S.R.); rcastro@ff.uc.pt (R.A.E.C.); antonio.evora@uc.pt (A.O.L.É.); troseiro@ci.uc.pt (T.M.R.M.); jcano@ci.uc.pt (J.C.)
[2] Faculdade de Farmácia, Universidade de Coimbra,3000–548 Coimbra, Portugal
[3] Ctr Quim Estrutural, Fac. Ciências, Universidade de Lisboa,1749-016 Lisboa, Portugal
[4] CfisUC, Departmento de Física, Universidade de Coimbra, 3004-535 Coimbra, Portugal; manuela@fis.uc.pt
* Correspondence: quierme@ci.uc.pt: Tel: +351 239854450

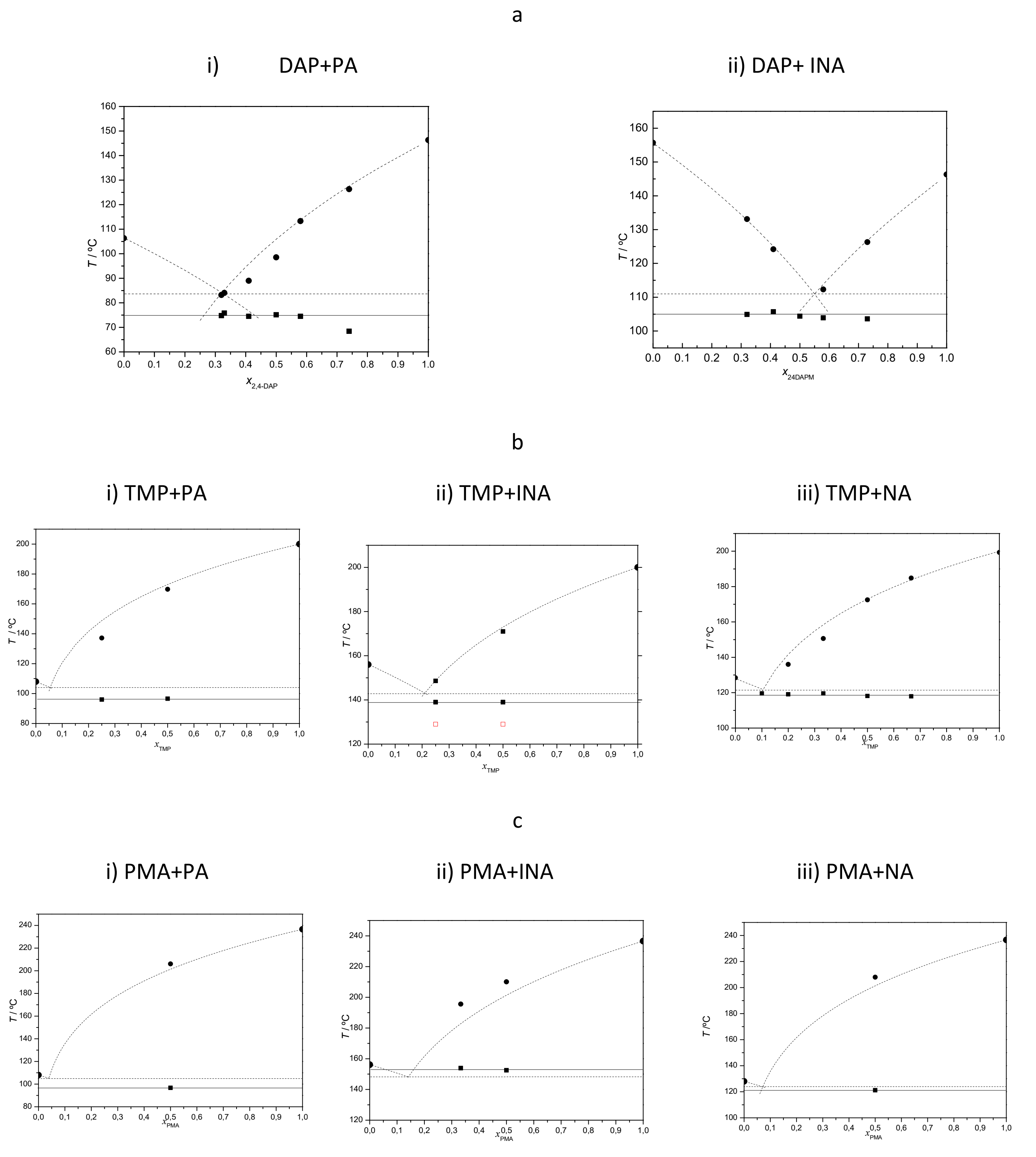

Figure S1. (Solid + liquid) binary phase diagrams of: a) i) DAP + PA), ii) DAP + INA); b) i) TMP + PA), ii) TMP + INA), iii) TMP + NA; c) i) PMA + PA), ii) PMA + INA), iii) PMA + NA. ■ and solid line - experimental *solidus*; ● - experimental *liquidus*. Dotted lines obtained using eq. (1).

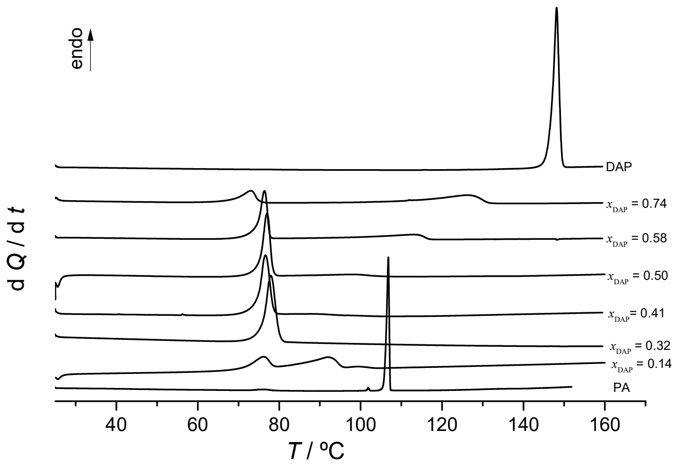

Figure S2. DSC heating curves of DAP + PA mixtures of different DAP mole fraction, x_{DAP}, prepared by LAG. β = 10 °C min[−1].

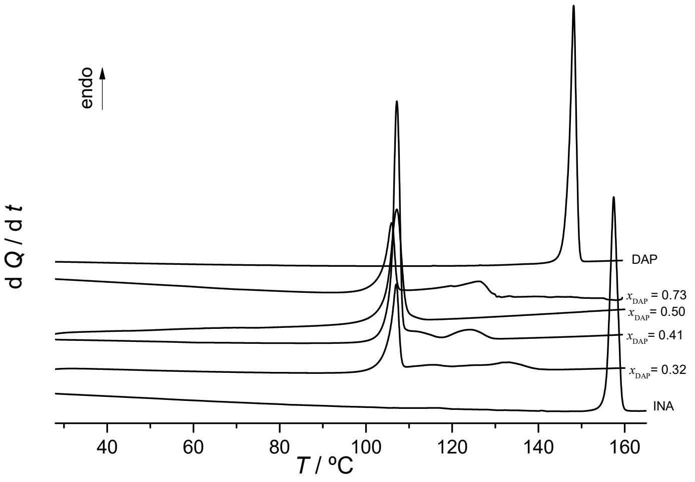

Figure S3. DSC heating curves of DAP + INA mixtures of different DAP mole fraction, x_{DAP}, prepared by LAG. β = 10 °C min[−1].

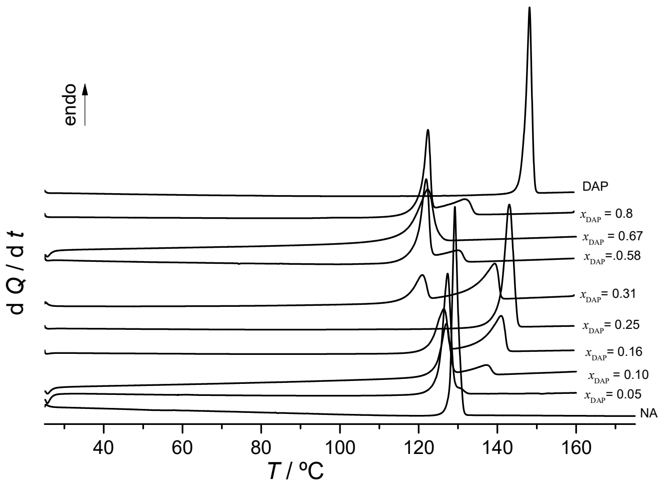

Figure S4. DSC heating curves of DAP + NA mixtures of different DAP mole fraction, x_{DAP}, prepared by LAG. β = 10 °C min^{-1}.

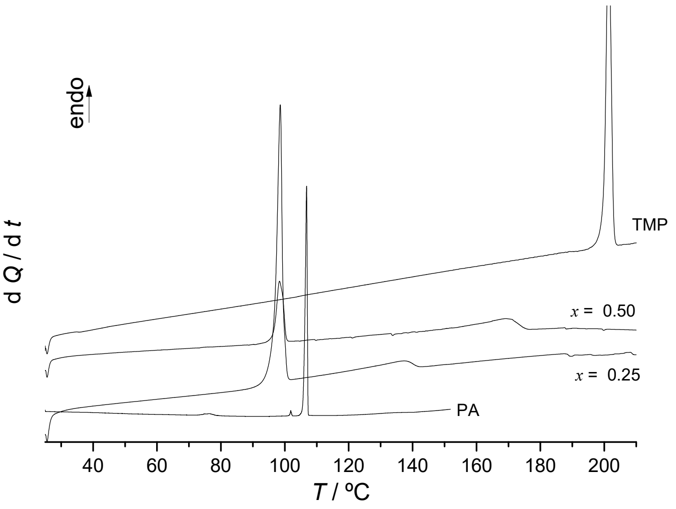

Figure S5. DSC heating curves of TMP + PA mixtures of different TMP mole fraction, *x*, prepared by LAG. β = 10 °C min^{-1}.

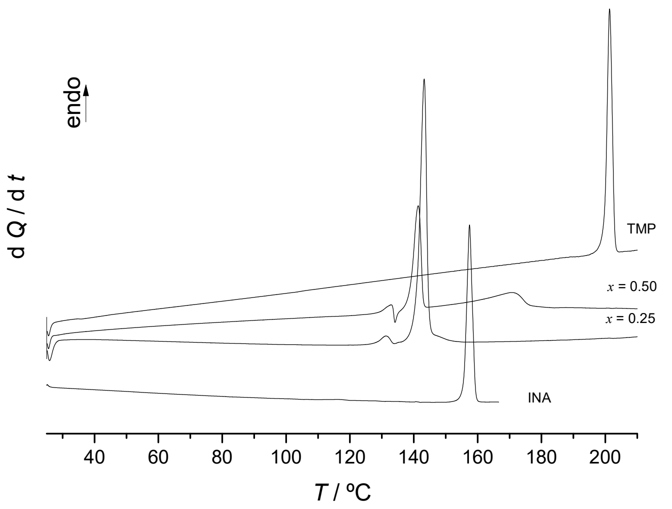

Figure S6. DSC heating curves of TMP + INA mixtures of different TMP mole fraction, *x*, prepared by LAG. $\beta = 10\ °C\ min^{-1}$.

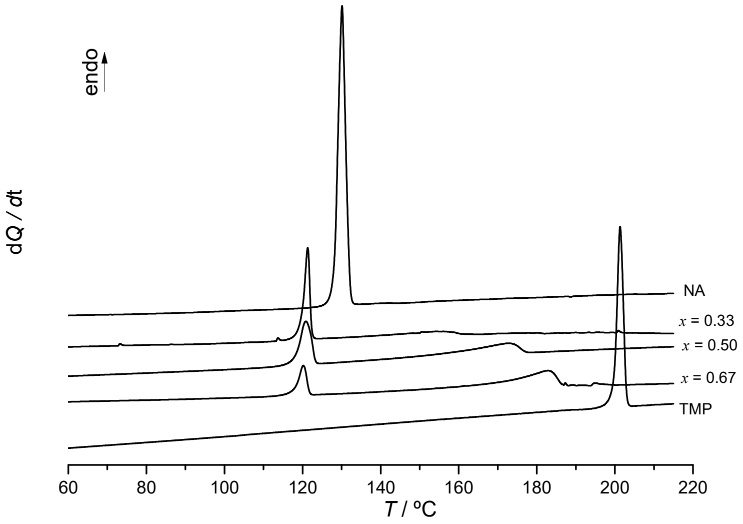

Figure S7. DSC heating curves of TMP + NA mixtures of different TMP mole fraction, *x*, prepared by LAG. $\beta = 10\ °C\ min^{-1}$.

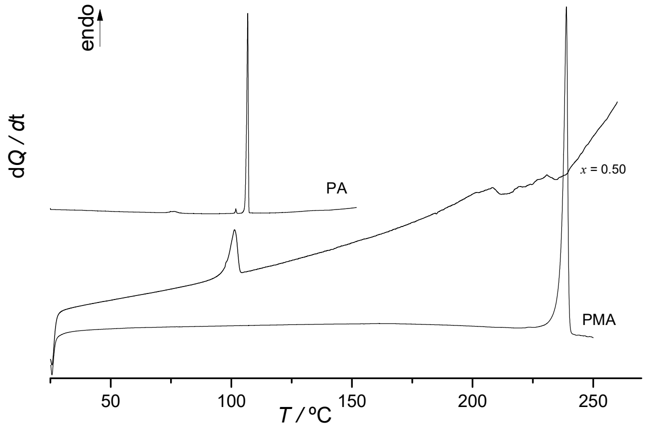

Figure S8. DSC heating curves of PMA + PA mixtures of different PMA mole fraction, *x*, prepared by LAG. β = 10 °C min^{-1}.

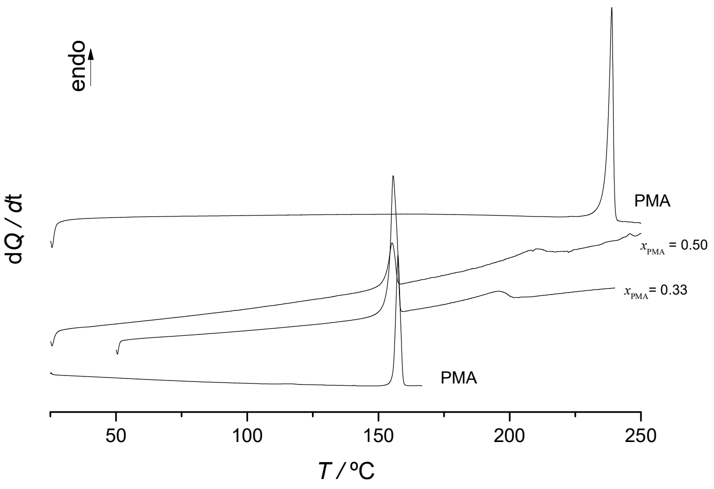

Figure S9. DSC heating curves of PMA + INA mixtures of different PMA mole fraction, *x*, prepared by LAG. β = 10 °C min^{-1}.

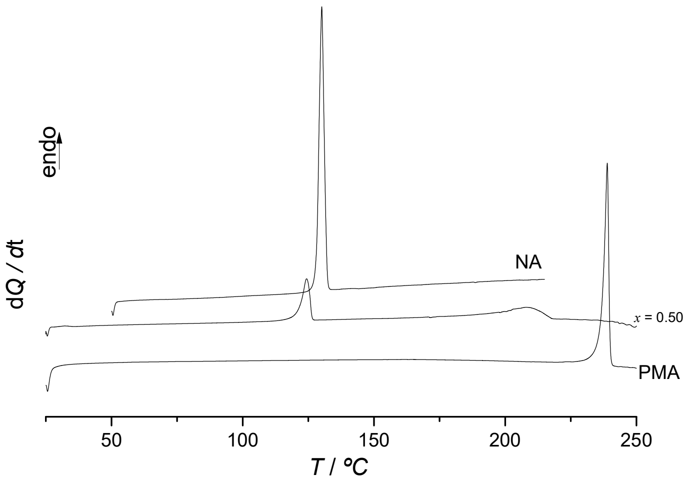

Figure S10. DSC heating curves of PMA + NA mixtures of different PMA mole fraction, x, prepared by LAG. β = 10 °C min^{-1}.

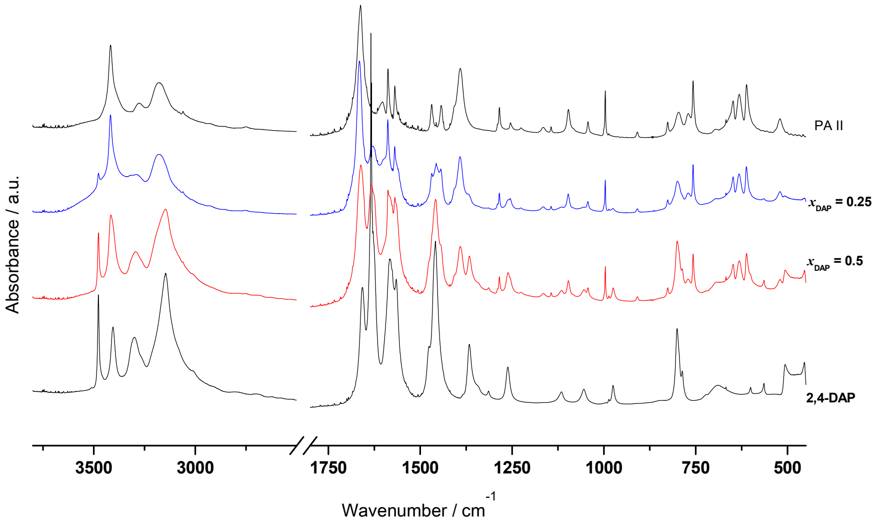

Figure S11. Infrared spectra of DAP + PA mixtures of different DAP mole fraction, x_{DAP}.

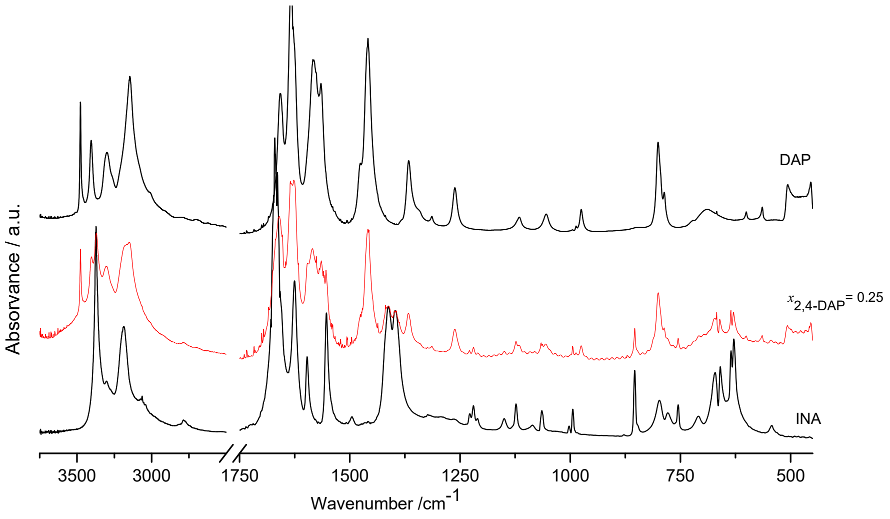

Figure S12. Infrared spectra of DAP+INA mixtures of different DAP mole fraction, x_{DAP}.

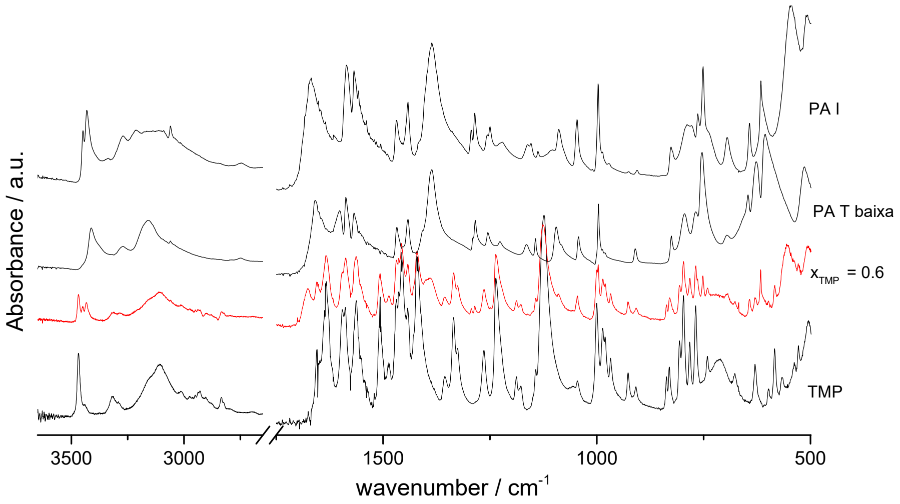

Figure S13. Infrared spectra of TMP+PA mixtures of different TMP mole fraction, x_{TMP}.

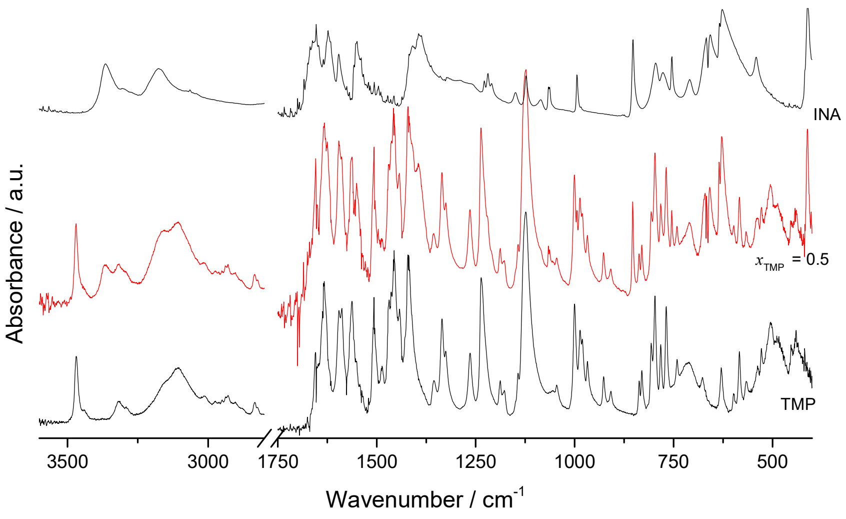

Figure S14. Infrared spectra of TMP+INA mixtures of different TMP mole fraction, x_{TMP}.

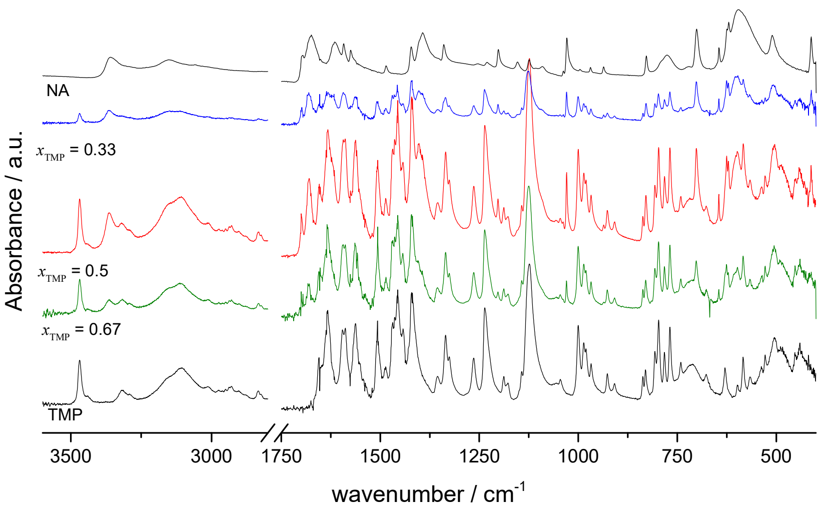

Figure S15. Infrared spectra of TMP+NA mixtures of different TMP mole fraction, x_{TMP}.

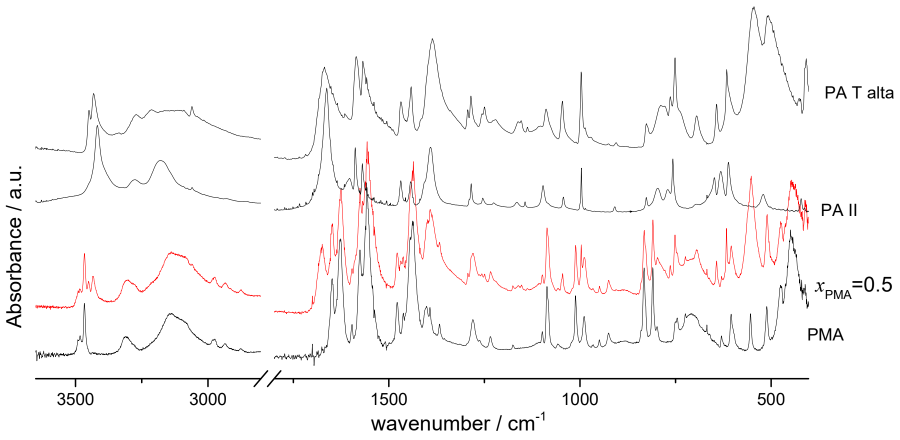

Figure S16. Infrared spectra of PMA + PA mixtures of different PMA mole fraction, x_{PMA}.

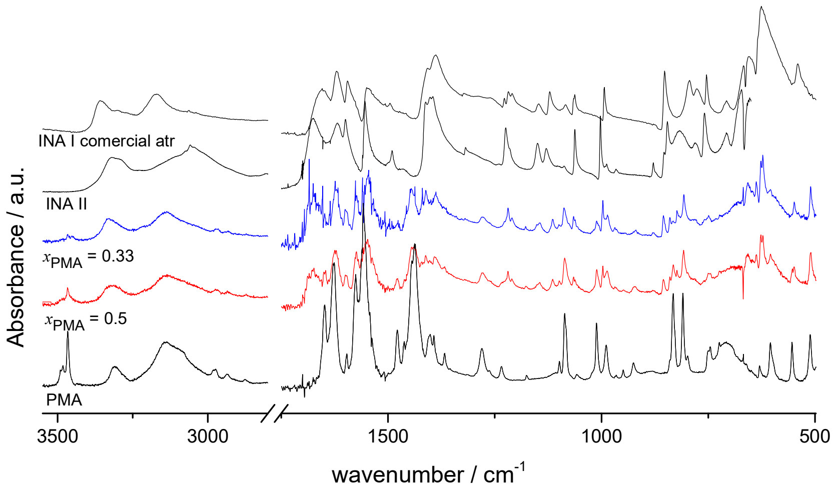

Figure S17. Infrared spectra of PMA + INA mixtures of different PMA mole fraction, x_{PMA}.

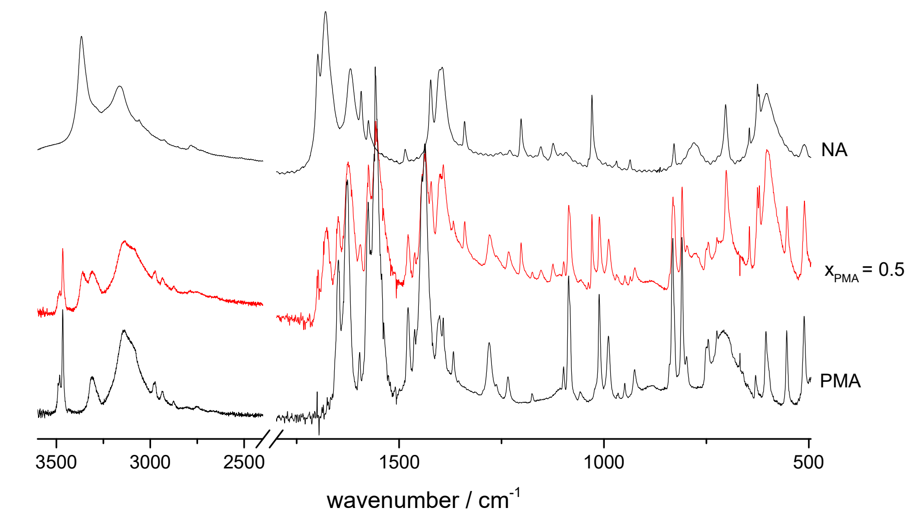

Figure S18. Infrared spectra of PMA+NA mixtures of different PMA mole fraction, x_{PMA}.

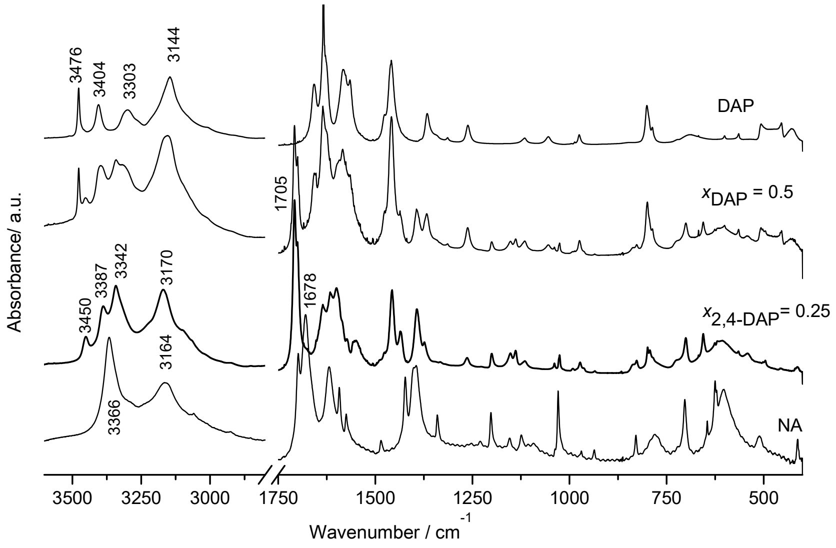

Figure S19. Infrared spectra of DAP+NA mixtures of different composition, obtained by LAG, ethanol assisted.

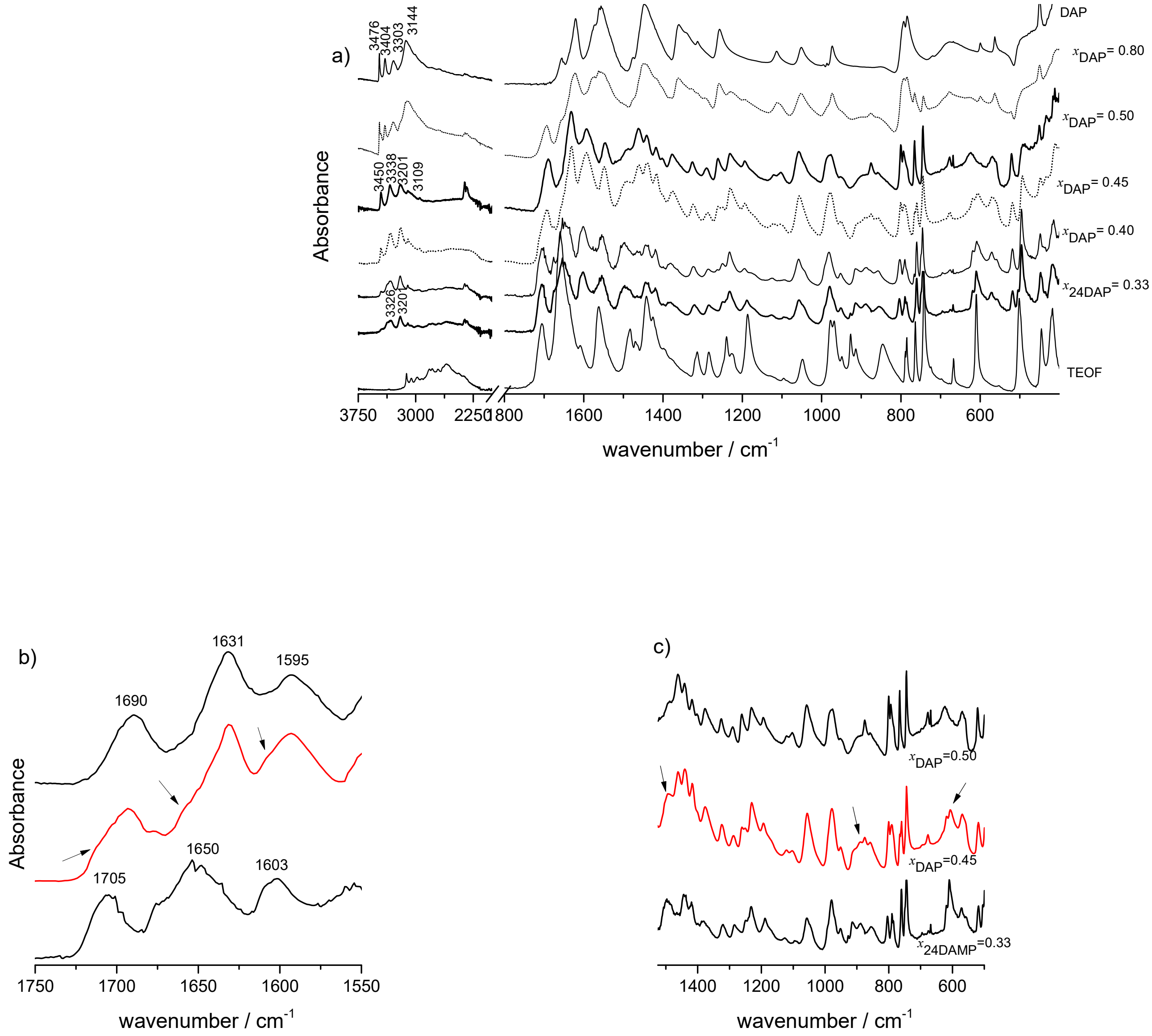

Figure S20. a) Infrared spectra of representative DAP+THEO mixtures prepared by ethanol assisted grinding. **b)** and **c)** detail of the spectra of mixtures of DAP mole fraction x_{DAP} = 0.50, 0.45 and 0.33.

Thermogravimetric curves obtained for the (1:2) DAP+THEO neat ground mixture using open pans and pierced ones, Figure S21, are different: two mass loss steps are observed in the first case and only one in the second one. The first mass loss step registered when using open pans is ascribable to sublimation with degradation starting at $T \approx 280$ °C.

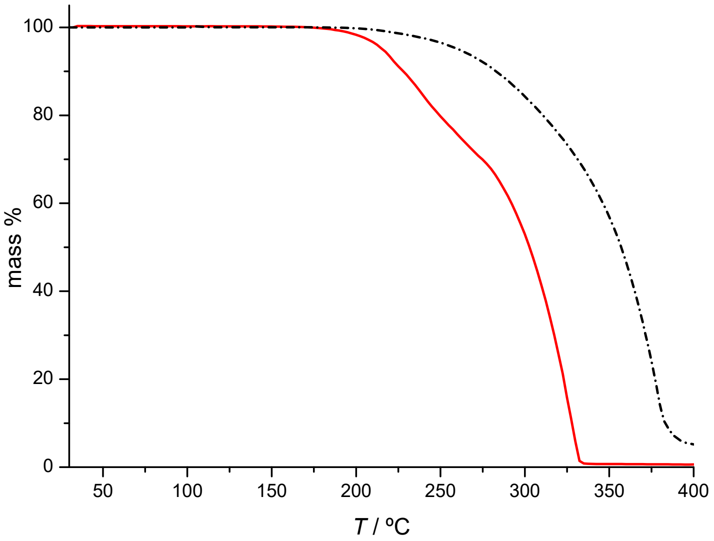

Figure S21. Thermogravimetric curves of DAP+THEO, x_{DAP} = 0.33, mixtures prepared by neat grinding obtained in open pans (red line) and in pierced pans (black dash-dot line).

a) b)

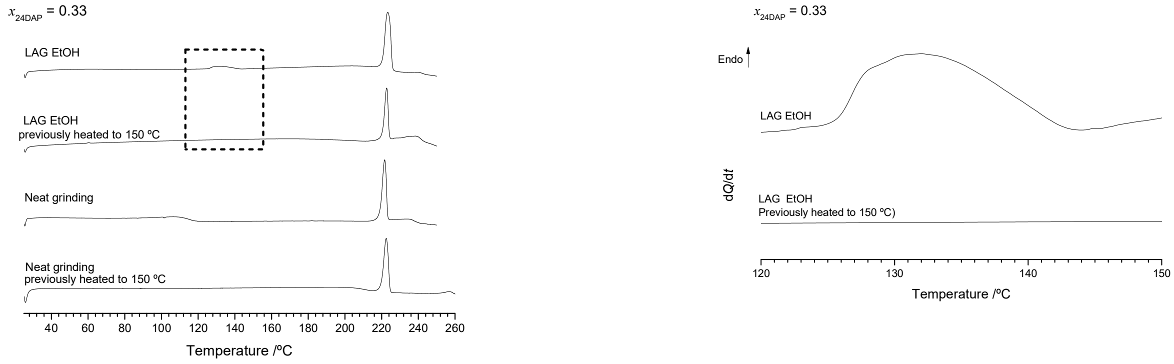

Figure S22. a).DSC heating curves of x_{DAP} = 0.33 mixtures prepared by LAG and by neat grinding, and submitted to different thermal treatments; **b)** expansion of the DSC thermograms in the zone indicated by the rectangle in Figure S20.a; β = 10 °C min^{-1}.

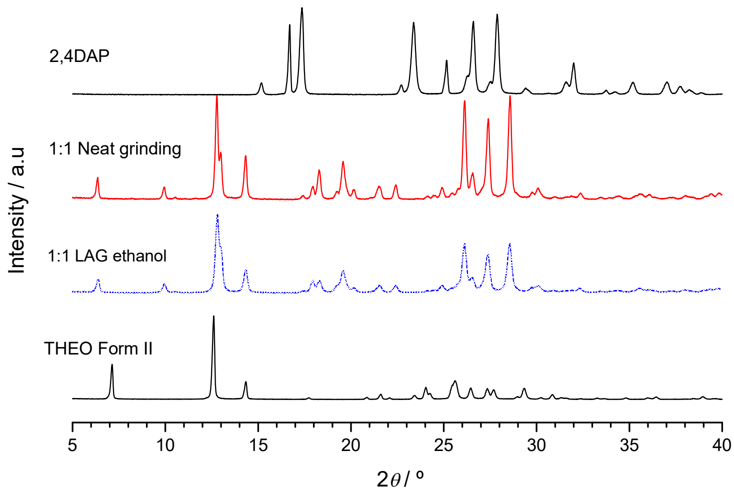

Figure S23. X-ray powder diffractograms of DAP+THEO (1:1) mixtures prepared by ethanol assisted grinding and by neat grinding.

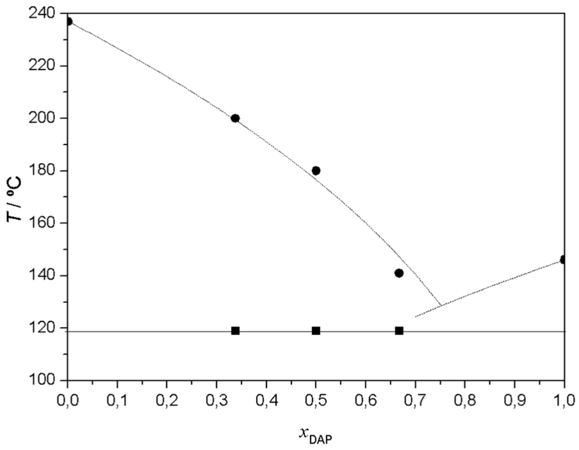

Figure S24. (Solid + liquid) binary phase diagram of DAP+CAF. ■ and solid line - experimental *solidus*; ● - experimental *liquidus*. Dotted lines obtained using eq. (1).

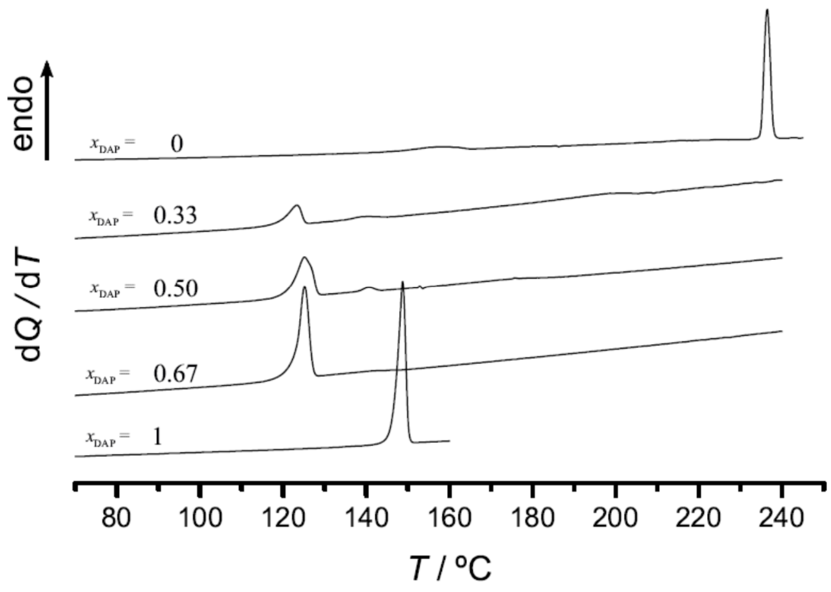

Figure S25. DSC heating curves of DAP+CAF mixtures of different DAP mole fraction, x_{DAP}, prepared by LAG. β = 10 °C min^{-1}.

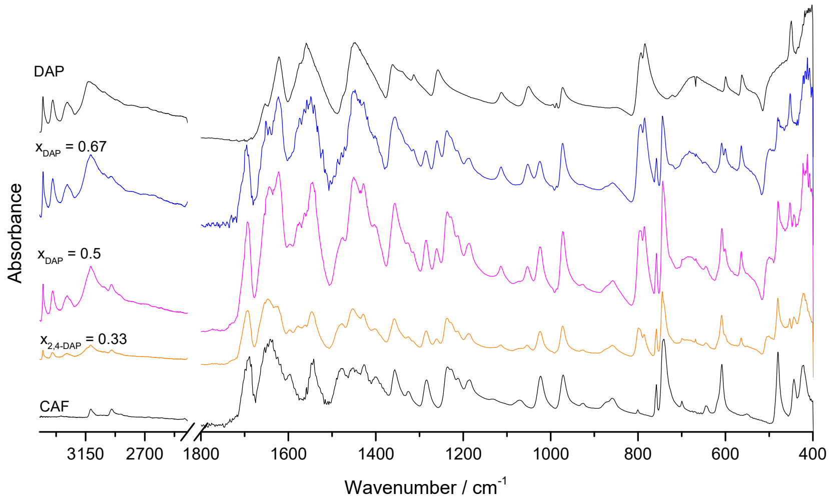

Figure S26. Infrared spectra of DAP +CAF mixtures of different DAP mole fraction, x_{DAP} prepared by grinding.

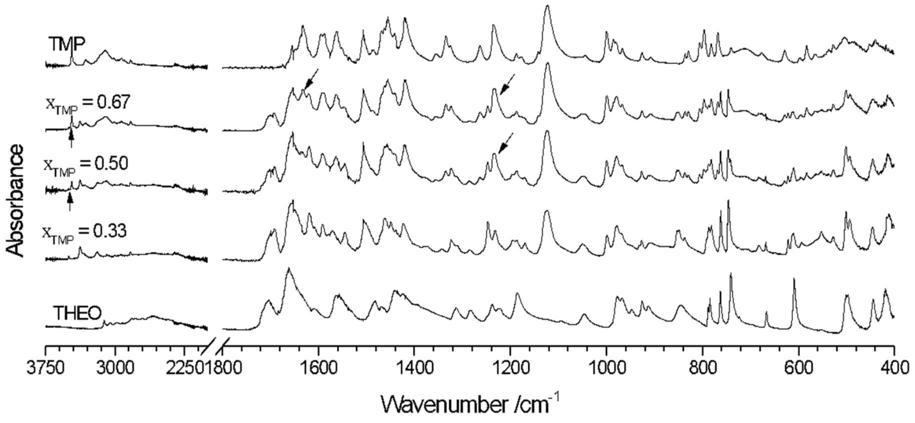

Figure S27. Infrared spectra of TMP+THEO mixtures of different composition, obtained by LAG, ethanol assisted.

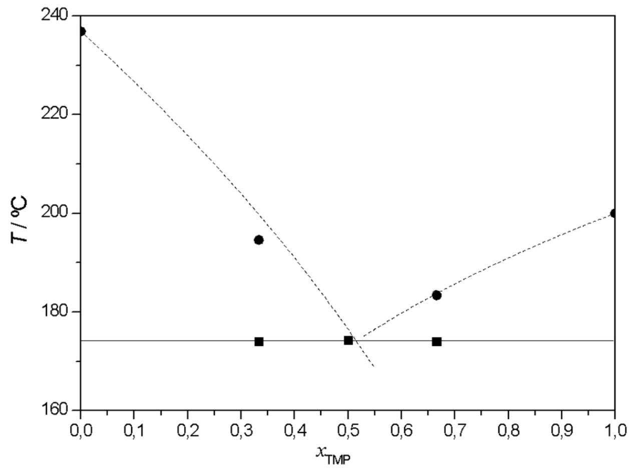

Figure S28. (Solid + liquid) binary phase diagrams of TMP+CAF. ■ and solid line - experimental *solidus*; ● - experimental *liquidus*. Dotted lines obtained using eq. (1).

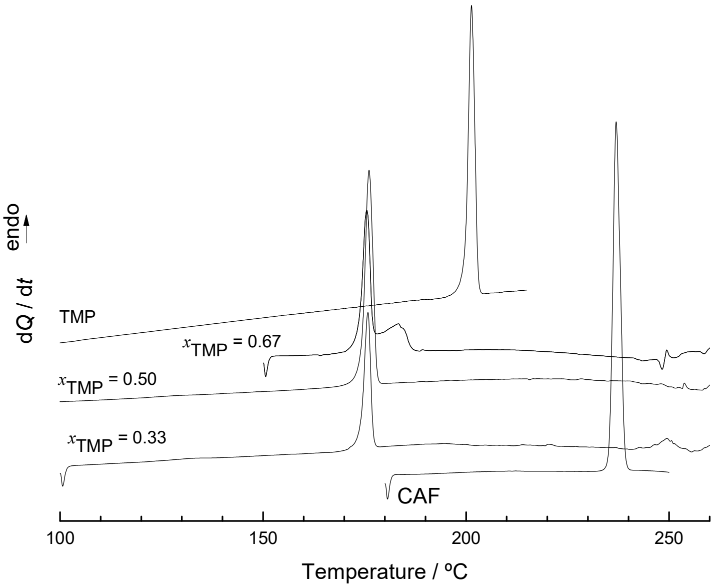

Figure S29. DSC heating curves of TMP + CAF mixtures of different TMP mole fraction, x_{TMP} prepared by LAG. β = 10 °C min$^{-1.}$

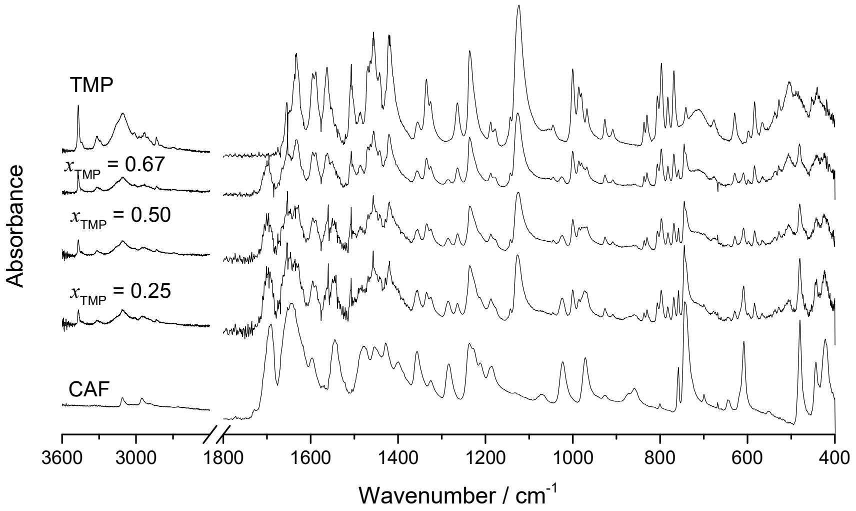

Figure S30. Infrared spectra of TMP+CAF mixtures of different TMP mole fraction, x_{TMP}, prepared by grinding.

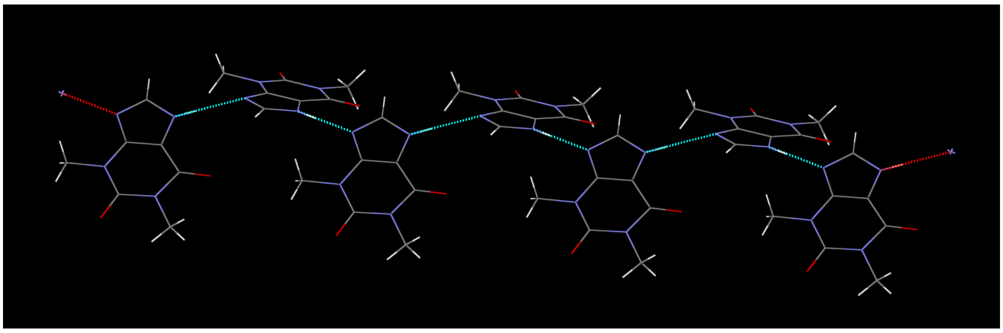

Figure S31. Hydrogen bonded chains joining the molecules of theophylline in polymorph II (CCDC 128707) [1].

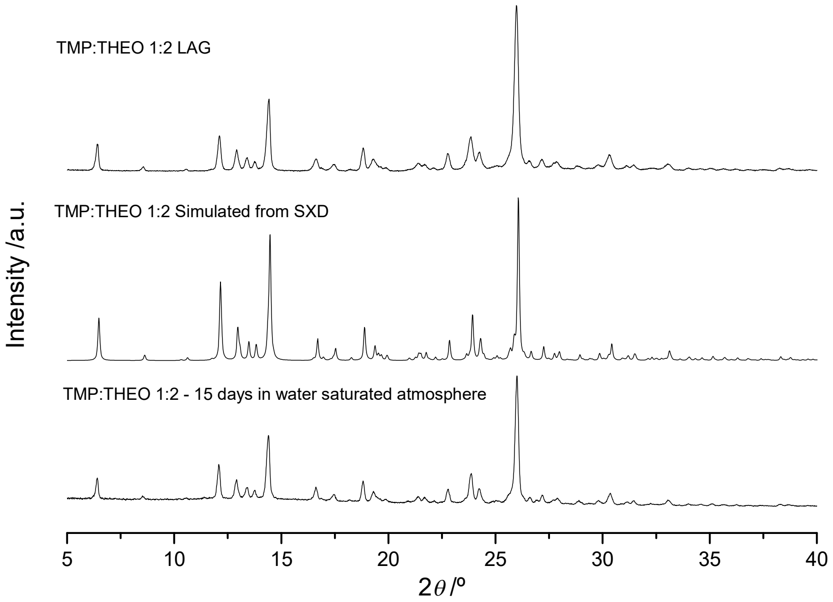

Figure S32. Comparison of X-ray powder diffractograms of the (1:2) TMP:THEO co-crystal obtained by LAG, simulated from CCDC2109486; and LAG co-crystal exposed to a saturated water atmosphere for 15 days.

Table S1. Melting temperature and enthalpy of the compounds investigated in this work.

Compound	T_{fus} / °C	$\Delta_{fus}H_m$ / kJ mol^{-1}
DAP	146.4 ± 0.3	22.9 ± 0.3
TMP, polymorph	199.3 ± 0.2	48.6 ± 0.4
PMA, polymorph I	236.6 ± 0.3	39.2 ± 0.3
PA, polymorph I	106.4 ± 0.4	19.4 ± 0.4
INA, polymorph I	155.5 ± 0.2	24.5 ± 0.3
NA, polymorph I	128.4 ± 0.3	25.5 ± 0.4
(1:3) DAP:NA co-crystal	141.4 ± 0.4	97 ± 1
CAF	236.9 ± 0.2	21.9 ± 0.3
THEO	270.2 ± 0.5	30.1 ± 0.4

Reference

1. Ebisuzaki, Y.; Boyle, P.D.; Smith, J.A. Methylxanthines .1. Anhydrous theophylline. *Acta Crystallogr. Sect. C* **1997**, *53*, 777–779.
